# Unraveling the key drivers of community composition in the agri-food trade network

**DOI:** 10.1038/s41598-023-41038-z

**Published:** 2023-08-26

**Authors:** Gian Paolo Clemente, Alessandra Cornaro, Francesco Della Corte

**Affiliations:** 1 Department of Mathematics for Economics, Financial and Actuarial Sciences, Università Cattolica del Sacro Cuore, Milan, Italy; 2 Department of Statistics and Quantitative Methods, University of Milano - Bicocca, Milan, Italy

**Keywords:** Applied mathematics, Environmental social sciences

## Abstract

In the complex global food system, the dynamics associated with international food trade have become crucial determinants of food security. In this paper, we employ a community detection approach along with a supervised learning technique to explore the evolution of communities in the agri-food trade network and to identify key factors influencing their composition. By leveraging a large dataset that includes both volume and monetary value of trades, we identify similarities between countries and uncover the primary drivers that shape trade dynamics over time. The analysis also takes into account the impact of evolving climate conditions on food production and trading. The results highlight how the network’s topological structure is continuously evolving, influencing the composition of communities over time. Alongside geographical proximity and geo-political relations, our analysis identifies sustainability, climate and food nutrition aspects as emerging factors that contribute to explaining trade relationships. These findings shed light on the intricate interactions within the global food trade system and provide valuable insights into the factors affecting its stability.

## Introduction

The analysis and deep understanding of the global food system are nowadays relevant topics. Indeed, the production of global food and agricultural products has increased significantly since the latter half of the previous century^1^. This process has been mainly driven by a combination of population and economic growth along with technological and cultural changes in production practices. Additionally, due to increases in population, wealth and urbanization, the world has seen an overall increase in food demand, coupled with a shift in dietary preferences towards more resource-intensive foods^2^.

In this context, new challenges emerge. Specifically, a deeply existential dilemma of the global food system has been emphasised^3^, such that at the same time we have the need of feeding a growing global population^4^ and also ensuring the sustainability of the natural resources and ecosystems^5^. On one hand, a food supply must be continuously guaranteed. On the other hand, current practices may have devastating consequences for natural environment^6^, thus undermining the foundation of the food system’s functioning. The agri-food system is indeed one of the primary drivers of global ecological changes^7^, while simultaneously being vulnerable to the impacts of climate destabilization^8^. There is also a a pressing need to ensure the sustainability of food production systems, which involves conserving biodiversity and protecting vital natural resources like soil, water, and forests. Agricultural practices, such as intensive monoculture, excessive use of pesticides and fertilisers, and overgrazing can exert a detrimental impact on the environment, leading to soil erosion, water pollution, and deforestation.

Therefore, it is imperative to move towards a sustainable and resilient food system that can effectively respond to evolving circumstances and emerging challenges. A rigorous and urgent food system transformation is required and science-based monitoring can play a pivotal role in guiding both public and private decisions, providing crucial support to decision-makers^9^. Food systems play a vital role in achieving the majority of the sustainable development goals set by the United Nations, as well as meeting the targets and commitments outlined in climate change conventions. Moreover, in addition to these global goals, food equity and food security^10^ emerges as a crucial aspect.

In this context, network analysis can be employed to investigate the structure and dynamics of the global food trade network, facilitating the identification of key players, trade relationships, and patterns of trade flows. Significant findings have been reported across various context. For instance, research has delved into the characteristics and evolution of the trade network concerning both the entire spectrum of agricultural products^11,12,13,14^ and specific commodities^15–18^. Additionally, studies have explored the virtual water network associated with agricultural products^19–23^ and the detection of community structures. Commmunity detection is a valuable tool for comprehending the structure and dynamics of the global food trade. It provides insights into patterns of regional specialization, the development of trade relationships, and the potential impacts of climate change and other external shocks on the global food system. For instance, community detection allows an analysis of how the current concentration of global food production in trade communities might be disrupted by climate change or other factors. It further enables prediction of the potential impacts of such disruptions on food security and trade. In the literature, several studies have yielded findings on trade patterns, community formation, and the dynamics of the global food system, as well as the potential related shocks^24–27^.

Furthermore, community detection has proven to be a relevant method for identifying clusters and structures within global food trade networks. This approach aids in revealing drivers of change, vulnerabilities and opportunities for policy intervention. Examining the drivers of global food trade entails considering economic, political, environmental, and social factors that influence trade relationships and food security. By doing so, strategies can be developed to mitigate the negative consequences of imbalanced or unsustainable trade patterns.

By pinpointing the drivers of changes in global food trade, such as price spikes and volatility, supply chain disruptions, and shifts in consumer demand, it becomes possible to anticipate and mitigate the adverse impacts of food insecurity, poverty, and environmental degradation. For example, trade agreements play a significant role in global food trade dynamics, activating new links and increasing traded volumes. Such agreements contribute to a more stable market and carry essential environmental implications in the realm of food trade^28,29^. Moreover, the study of drivers in global food trade can inform efforts to promote sustainable agriculture practices, reduce waste and spoilage, and ensure equitable access to nutritious food for all^30,31^. Integrating social, economic, and environmental considerations into the analysis of global food trade allows for the development of more resilient and inclusive food systems capable of withstanding shocks and adapting to changing circumstances.

In this context, our paper shows how the implementation of community detection methods can reveal important attributes of the agri-food trade network, providing valuable insights into the drivers that impact global food trade. To address this matter, we employ a combination of network theory and supervised learning methodologyutilizing the food and agricultural trade dataset collected by Food and Agriculture Organization of the United Nations (FAO). The data^32^ encompass volumes and values of imports/exports exchanged annually by all countries worldwide, regarding all food and agricultural products, spanning the period from 1986 to 2020.

Using this data, we build temporal directed and weighted networks, with nodes representing countries and directed weighted edges reflecting either the volume or the monetary value of trades between pairs of countries in a specific year. Consequently, we conduct a preliminary analysis of the trade network’s topology evolution. It is noteworthy that our analysis is employed by considering the aggregate total flow, achieved through the aggregation of various types of commodities involved. This approach enables us to offer an overview of global trade patterns and identify factors that, on average, influence the behaviour of trade in agri-food network. However, as highlighted in the literature^24,27^, adopting a multi-network approach, differentiated by commodities, may allow for a more granular analysis. A commodity-level approach can reveal specific trade communities and relationships within individual food product categories, capturing the heterogeneity of commodity-specific networks.

As a second step, for each year, we identify communities of countries by applying the InfoMap methodology^33,34^. Unlike the classical modularity maximization approach^35–38^, this flow-based and information-theoretic method allows to consider patterns of flows in the network, which is a crucial aspect when dealing with trades. The partitioning is based on the flow induced by the connections pattern in a given network. Analysing the evolution of these communities allows to depict how countries’ connections evolved in the global food system.

Indeed, the structure and dynamics of the agri-food trade network can have important economic, social, and environmental implications. For example, alterations in trade policies or market conditions can lead to ripple effects throughout the network, impacting the livelihoods of farmers, producers, and other stakeholders. Additionally, the transportation and distribution of food products within the network can give rise to significant environmental impacts, including greenhouse gas emissions and land use changes.

Therefore, the identification of communities marks the initial step towards comprehending the structure of a complex system. In a final step, we shift our focus to characterising the communities based on the shared attributes among the elements within each community. Evaluating the results of a community detection method encompasses various approaches, but characterization often proves to be a challenging task due to the potential heterogeneity among system elements^39^. Consequently, we adopt a multi-step procedure. Firstly, we associate a set of attributes to each node, enfolding various characteristics of the country, including economic, demographic, social, geographical and meteorological aspects. Secondly, we employ a random forest methodology^40–42^ to identify the variable importance^40^ in explaining the composition of communities. Finally, we combine the communities obtained from the InfoMap approach with the relevant variables selected via the random forest. This combination enables us to measure the over-expression^39^ of relevant attributes within each community and across different time periods. Through this proposed methodology, we can analyse the evolution of communities over time in the global agri-food system and identify the primary drivers that have guided this evolution.

At a preliminary level, the results confirm the significant evolution of both the number of trade partners and the trade value in the agri-food network over time^14^. In the early 20th century, most countries relied on domestic food production to fulfil their needs, and international trade in agricultural products was relatively limited. However, with the growth of global transportation and communication networks, coupled with the liberalization of trade policies, agri-food trade experienced a remarkable expansion. Presently, a majority of countries engage in agri-food trade, with some specializing in the production of specific crops or livestock products. Over time, the number of trading partners has also risen, with many countries now importing and exporting a wide range of agricultural products.

Based on the analysis of community composition, a notable overlap is observed between the communities derived from monetary values and those obtained from trade volume. However, when focusing on trade volume, a more pronounced decomposition into stronger subgroups becomes apparent. This finding suggests that the formation of communities in food trade is significantly influenced by geo-political factors. The interconnectedness of countries through trade agreements, regional alliances, and shared economic interests plays a crucial role in shaping these communities. As a result, geo-political trade dynamics can lead to the development of regional trade blocs and partnerships, where countries within the same geographic area or with similar political ideologies tend to trade more frequently with each other. In particular, consistent with other findings in the literature^26^, our analysis reveals a noticeable trend of increasingly closer trade relationships among member states of the European Union over time, indicating the formation of a cohesive single community. The effects of this evolution are also reflected on the community composition involving the former Soviet Union and the Eastern European countries. In the past, the Soviet Union and Eastern European countries shared strong political and economic bonds, leading to the establishment of a closely integrated trade community. However, with the dissolution of the Soviet Union and subsequent political changes, the dynamics of this community have undergone significant transformation. Currently, we find a community formed by Russia, Central Asian, and Caucasian countries, due to the historical connections and geographic proximity. Over the extended time period under consideration, the members of the North American Free Trade Agreement (NAFTA, now USMCA since July 2020) are observed to form a cohesive community alongside China, Japan, members of Association of Southeast Asian Nations (ASEAN) and Australia, aligning with earlier findings^26^. This outcome highlights the significant connections between these nations at an aggregate level. However, it is worth noting that studies in the literature that delve into product-level analysis^27^ or focus on virtual water trade values^25^ often reveal that China, Japan, Australia typically belong to distinct communities, revolving around other communities, such as the North American, South American, and Russian ones. This indicates that while the broader trade relationships show a shared community, the intricacies at a commodity level uncover different trade patterns and partnerships among these countries. Additionally, we notice a persistent Middle East community, including most of the members of the Arab Free Trade Area. The importance of geopolitical aspects in the Middle East community cannot be overstated. The region’s complex history, diverse cultures, and strategic location have made it a focal point for global trade and political interactions for millennia.

In examining the factors that influence community compositions, in line with the literature^28^, geographical proximity plays a significant role in shaping the structure and dynamics of communities in various systems. However, although the results reflect the influence of distance on the trade structure, other drivers also appear relevant. The composition of specific communities is influenced by similarities in topological indicators, such as degree and strength. When entities within a network exhibit comparable degrees (number of connections) or strengths (weighted connections), they tend to gravitate towards forming cohesive communities within the network. This similarity in connectivity patterns enhances the likelihood of interactions and shared characteristics, ultimately shaping the community structures observed in the network. We also find that the presence of similarity in political attributes, particularly in terms of economic freedom and government governance, exerts a substantial influence on the bilateral trade of food products^14,43^. Countries that share negative political stability tend to belong to the same community, , suggesting that political factors significantly impact trade relationships. Finally, agri-food trade remains subject to fluctuations in global markets and changes in policies, as well as disruptions. In this context, we find how the environmental and social aspects have emerged as factors in explaining factors trade relationships in recent years.

The remaining sections are organised as follows. We describe the methodology applied and the data used. Then, main results are displayed together with insights and practical implications. A short conclusion on main findings follows. Additional figures and results are devoted to the Supplementary Material.

## Methods

We provide here a brief description of the methodology used for analysing the evolution of the agri-food network and for identifying and characterising main communities. Specifically, we follow a three-step procedure, which will be detailed in the next subsections. Firstly, we construct a temporal directed and weighted network. Next, we conduct a preliminary analysis of the topology of the network over time and subsequently apply the InfoMap methodology for detecting communities in each time period. Finally, we employ a random forest approach to select the importance of node attributes in explaining the communities composition. Then, we measure the over-expression of relevant attributes in the different groups.

### Agri-food network

We consider a temporal directed and weighted agri-food network ${\mathscr {G}}$, where the relationships between nodes are characterised by the presence of temporal, directed, and weighted edges. This type of network allows to represent systems that evolve over time, such as trade. Temporal edges represent one-way links between two nodes and indicate the presence of a connection at a specific time, highlighting the temporal dimension of the network. The intensity of the connection is then captured by the weight of the edge.

We consider a number of time periods *T*, with $t=1,...,T$. The agri-food network ${\mathscr {G}}$ can be represented as a collection of directed and weighted networks $G_{t}=(V_{t},E_{t}, w_{t})$ where $V_{t}$ is the set of $n_{t}$ nodes, $E_{t}$ is a subset of $V_{t}\times V_{t}$ (set of $m_{t}$ directed edges) and $w_{t}$ is a real positive weight assigned to each edge. Each graph will be described by a real $n_{t}$-square matrix ${\textbf{W}}_{t}$ (the weighted adjacency matrix) whose entries are $w_{ij,t}>0$ if $(i,j)\in E_{t}$, and 0 otherwise. In this analysis, each node represents a country and the entry $w_{ij,t}$ will signify the weighted edge from node *i* to node *j*. In other words, $w_{ij,t}$ will denote either the volume or the monetary value of export from country *i* to country *j* at time *t*.

For each time period, we analyse the topological structure of the network using classical global indicators, including average in- and out-degree^44^, average in- and out-strength^44^, density^44^, average directed clustering coefficients^45,46^. Additionally, as explained in the numerical section, local topological indicator, as degree, strength, directed clustering coefficient, hub and authority scores^47,48^ will be used as node attributes.

### Community detection

On each network $G_{t}$, we apply the InfoMap^33,49^ method for detecting communities. Like other community detection methodologies^50,51^, the goal is to identify communities in a network addedthat exhibit denser connections within themselves than with the rest of the network. The uniqueness of this method lies in optimizing an objective function based on the so-called map equation.

The InfoMap algorithm exploits the duality between identifying community structures and minimizing the description length of the motion of a so-called random walk^52^. The InfoMap algorithm starts by partitioning the network into a set of modules or communities, where each module comprises nodes tightly connected to each other and weakly connected with the rest of the network. Subsequently, the algorithm seeks to minimise the InfoMap cost, a quantity that measures the information required to encode random walks on the network. The cost is based on the idea that random walks on the network are more likely to remain within a module rather than to jump to another module. Therefore by assigning shorter codes to nodes within the same module, we can minimise the amount of information needed to describe the random walk.

The community structure is iteratively refined by merging or splitting modules based on the InfoMap cost. In each iteration, the algorithm assesses the cost of merging two modules, splitting a module, or maintaining the current module structure unchanged. The process continues until the cost can no longer be reduced by merging or splitting modules. This method is powerful and flexible, with one of its advantages being its scalability, allowing it to be applied to very large networks with millions of nodes and edges.

Through this approach we identify in each time period a number of non-overlapping communities $g_{t}$ and a set $C_{t}=\{C_{t,1},...,C_{g_{t},t}\}$ that include each community $C_{t,k}$ with $k=1,...,g_{t}$. Each community $C_{t,k}$ consists of a group of nodes, with the number of nodes defined as $n_{C_{t,k}}$ and it holds that $\sum _{k=1}^{g_{t}}n_{C_{t,k}}=n_{t}$.

### Identifying relevant attributes

In each time period, we enrich each network $G_{t}$ by considering a set of node-attributes. Specifically, we now have a directed and weighted attributed graph, $G^{F}_{t}=(V_{t},E_{t},w_{t},F_{t})$, where $F_{t}=\left\{ F_{t}^1,\ldots ,F_{t}^{a}\right\}$ is a set comprising *a* attributes. $F_{t}^h=\left\{ f^h_{t,1},\ldots ,f^h_{t,n_{t}}\right\}$ with $h=1,\dots ,a$, represents the value of the feature *h* at time *t* for each node.

As detailed in the numerical section, these attributes encompass local topological indicators computed at node level as well as countries data related to geographical, economic, demographic, sustainability and meteorological aspects. The objective is to assess the relevance of these attributes in explaining the classification induced by the InfoMap procedure and obtained in the previous step.

To achieve this, we apply a random forest methodology^40,53,54^ to predict the community to which a country belongs based on a set of independent variables (i.e. the attributes). Following a standard approach, we divide the data into a training set and a testing set using a classical split of 70 and 30%, respectively. The algorithm builds a collection of decision trees using random subsets of the training data and predictors, and then combines the predictions of the trees to assign a class label to each observation. The model’s performance has been evaluated using a testing set and optimised by adjusting the parameters of the algorithm.

To enhance the performance of the model and avoid overfitting or underfitting, we optimise the number of variables considered. We achieve this through both a grid search algorithm^55^ and the minimization of the Out-of-bag error^40^. Subsequently, we evaluate the model’s performance through classical measures, such as the the confusion matrix and the level of accuracy.

After selecting the model, we focus on variable importance, which measures the relative importance of each feature in determining the output. For this purpose, we compare the results of alternative methods^40^ based on mean decrease impurity (MDI) and mean decrease accuracy (MDA). MDI represents the average reduction in impurity (measured by the Gini index) across all trees in the forest when a particular variable is used for splitting nodes. On the other hand, MDA measures the average decrease in accuracy of the model when a particular variable is permuted or randomly shuffled across all samples in the test set. It is worth noting that the variable importance scores produced by mean decrease impurity can be biased^56,57^ towards variables that have a larger number of unique values or levels, as these variables are more likely to be selected for splitting. To mitigate this bias, we also apply permutation feature^40^ importance as an alternative method. It involves permuting the values of a variable and measuring the change in the model’s performance before and after the permutation. The importance of a variable is measured as the average decrease in performance over multiple permutations.

### Characterizing communities

The classification into communities is just the initial step in understanding the complex of the agri-food network. It is essential to interpret these communities further. Therefore, we aim to characterise the groups obtained by measuring the over-expression of the relevant features derived in the previous step, combining the results from both the InfoMap and Random Forest approaches.

We follow the approach proposed in Tumminello et al.^39^. For a specific time period *t* and neglecting for the sake of simplicity the subscript *t*, we transform each feature $F^{h}$ into five classes resulting in categories $d=1,...,5$. For a given community $C_{k}$, obtained in the same time period, and for one of the *d* categories of the feature $F^{h}$, we have the following quantities; $n_{C_{k}}$(number of nodes in the community), $n_{F^{h}_{d}}$ (number of nodes that belong to the category *d* for the attribute $F^{h}$), and $n_{F^{h}_{d},C_{k}}$ (number of nodes in the community that also belong to the category *d* for the attribute $F^{h}$).

With these values, we compute the probability of observing a number $n_{F^{h}_{d},C_{k}}$ using the following formula:

$$\begin{aligned} P\left( n_{F^{h}_{d},C_{k}}\right) =1- \sum _{x=0}^{n_{F^{h}_{d},C_{k}}-1}P_{H}(X=x)= 1-\sum _{x=0}^{n_{F^{h}_{d},C_{k}}-1} \frac{\left( {\begin{array}{c}\displaystyle n_{C_{k}}\\ \displaystyle x\end{array}}\right) \left( {\begin{array}{c}\displaystyle n-n_{C_{k}}\\ \displaystyle n_{F^{h}_{d}}-x\end{array}}\right) }{\left( {\begin{array}{c}\displaystyle n\\ \displaystyle n_{F^{h}_{d}}\end{array}}\right) } \end{aligned}$$

where $P_{H}(X=x)$ is the probability mass function of a hypergeometric distribution with parameters *n* (population size), $n_{C_{k}}$ (number of success states in the population, and $n_{F^{h}_{d}}$ (number of draws). As shown in Tumminello et al.^39^, the resulting probability can be compared with a *p* value, and the statistical threshold for multiple hypothesis tests can be adjusted using the Bonferroni correction^58^. The process is repeated for each time period, community and relevant feature.

## Results and discussion

Here, we provide an overview of the procedures employed to construct the dataset and the temporal network. Additionally, we emphasise the primary insights gleaned from a preliminary analysis from a preliminary analysis of the network’s behaviour over time and the identification of key players within the network. Next, our attention shifts to the communities identified through the InfoMap methodology, and we delve into characterising these communities using both the results obtained from the random forest methodology and an analysis of the over-expression of the main variables within the detected communities.

### Preliminary analysis

We begin by examining the annual imports and exports of all countries worldwide for food and agricultural products during the period from 1986 to 2020. To ensure the reliability of the data, we apply an algorithm^59,60^ to create a consistent and homogenous data source, addressing certain inconsistencies arising from importers’ and exporters’ declarations. The dataset covers over 200 countries and includes more than 300 different agri-food commodities present in the data.

**Figure 1:**
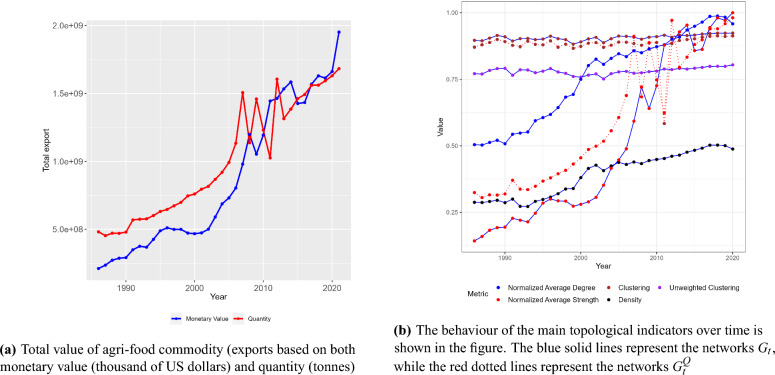
Total export and network topological indicators over time.

The monetary value of global agricultural exports has more than tripled since the beginning of the century, as depicted in Fig. 1a. This trend aligns with findings in the literature^26^. According to FAO^61^, food exports have accounted for a larger share of the total agricultural trade during the same period. The rapid growth observed between 2000 and 2010 can be largely attributed to higher commodity prices. The fluctuations in export values closely mirror the international price trends, particularly evident during the food security crises of 2007–2008, when record high prices for cereals were reached. This period also saw an expansion in biofuel consumption, increased energy prices, relative price effects associated with a weaker US dollar, and shifts in consumption patterns in emerging economies like China, favouring high-value products such as meat and dairy^62^. In 2013–2014, there was a decline in prices, primarily affecting cereals and oilseed products, due to a large global harvests and a slowdown in the biofuel demand. Nonetheless, trade values and volumes have continued to increase. During the coronavirus pandemic in 2020, while the overall trade in manufactured goods suffered, food exports were relatively less affected. Furthermore, an increasing pattern is noticeable in terms of the quantity of exports and imports. However, the disparity between these two behaviours, measured in US dollars and tonnes, respectively, underscores the impact of the price patterns mentioned earlier.

Using this dataset, we build a directed and weighted temporal network, where, in each time period *t*, a network $G_{t}$ is obtained. In this network, individual countries are nodes and trade between any two countries is represented as an edge with an associated weight, which is defined as the monetary value of the trade export flows in US dollars. Throughout our global analysis, we have 35 different networks spanning the years from 1986 to 2020. We consider roughly 200 countries (for a complete list of countries see Supplementary Material, Section 1.1)﻿ and around 20,000 edges (see Fig. 1 in Supplementary Material, Section 1.2 for a representation of the network in 2020). However, it is important to note that the number of countries in our analysis may vary over time due to changes in political boundaries. Nonetheless this variation does not affect the significance of our results, as the set of countries remains consistent in each year and each country-pair represents an observation of a specific year. It is worth mentioning that while other contributions in the literature^12,24,26,27^ focus on analysing the value of trade, it is essential to acknowledge that monetary value alone may not fully capture critical aspects such as food availability, access, and use, which are vital for comprehending the dynamics of the network. Therefore, to further enrich our analysis, we apply the proposed methodology to a second directed and weighted temporal network. In each time period, the network, denoted as $G^{Q}_{t}$, has the same size and order of $G_{t}$ but the weights of directed edges are defined as the quantities of trade in tonnes (for a visualization of the network in 2020, refer to Figure in Supplementary Material, Section 1.2).

Upon focusing on a preliminary analysis of the topology of the binary network over time, we notice a higher connectivity in the network. This is due to the fact that the average number of connections, and consequently the number of trade partners, has increased over time (see Fig. 1b). In the beginning, the network was not very dense (0.28 in 1986), but countries have progressively diversified their partners, resulting in a higher density (almost 0.5 in 2020, see Fig. 1b). When introducing weights, a significant increase in the trade intensity is evident for both monetary values and food trade relations, confirming previous findings^14^. Additionally, the network shows a high level of clustering coefficient^46^ in both weighted and unweighted cases. While not shown in Fig. 1b, directed patterns^45,46^ also exhibit high levels for both imports and exports, indicating the presence of strong triads of countries engaging in mutual trade relationships in the networks $G_{t}$ and $G^{Q}_{t}$. This preliminary analysis of the topology of the network confirms that the global food and agricultural trade network has become denser^12,15,19,22,23^ over time, with more countries engaging in trade, forming triads^63^ and involving a greater participation from low- and middle-income countries. One of the key drivers behind this process of globalization is trade liberalization at the multilateral and regional levels^64^.

**Figure 2:**
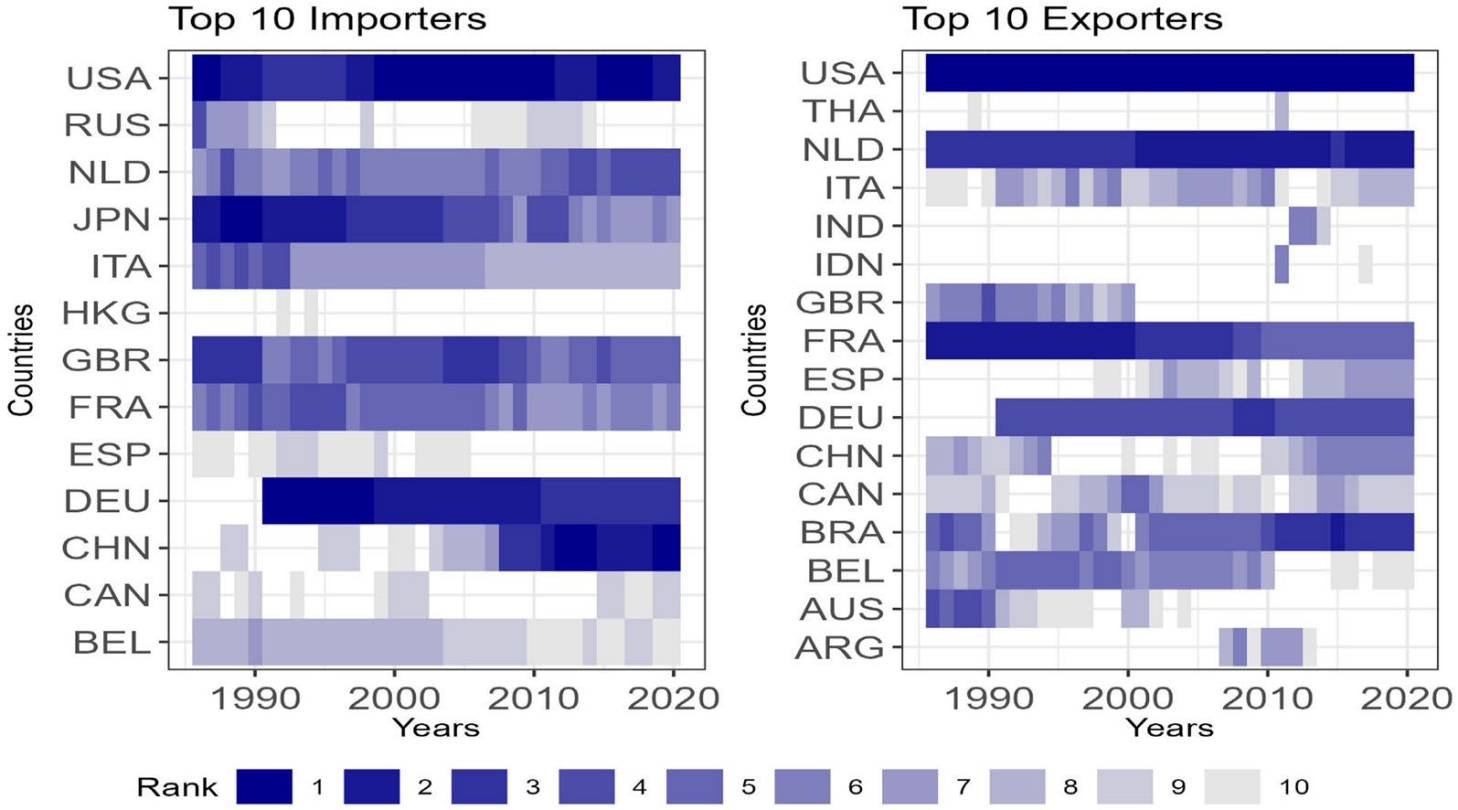
Evolution of ranking of top ten exporters and importers over time based on monetary value. Countries included in the top ten for at least one of the analysed years are displayed. If a country’s rank falls below the top ten in a specific year, an empty value is shown.

**Figure 3:**
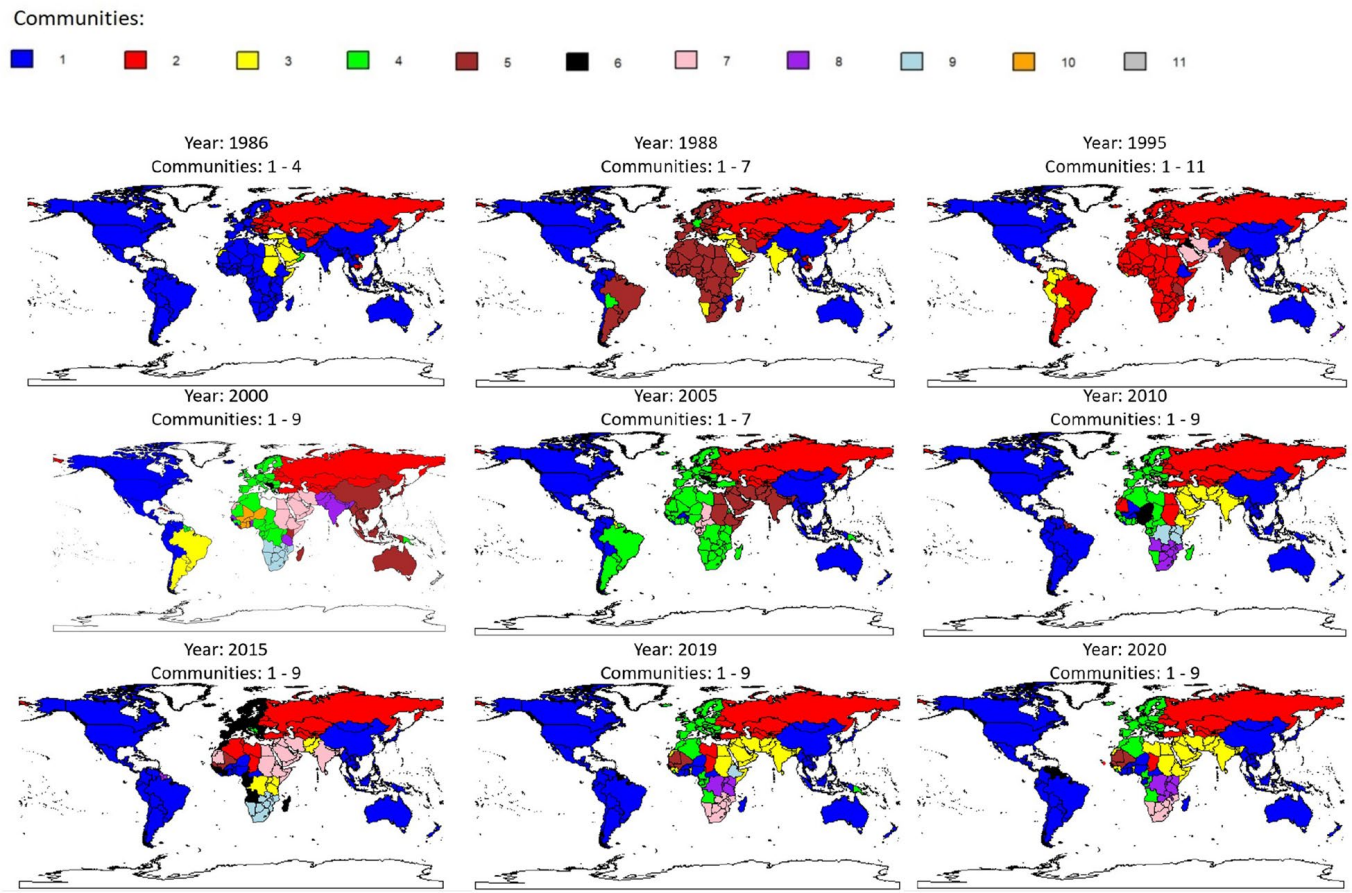
Communities detected via InfoMap approach on the networks $G_{t}$. Number of communities detected for each year are reported above each Figure. The maps were generated using R software^78^.

**Figure 4:**
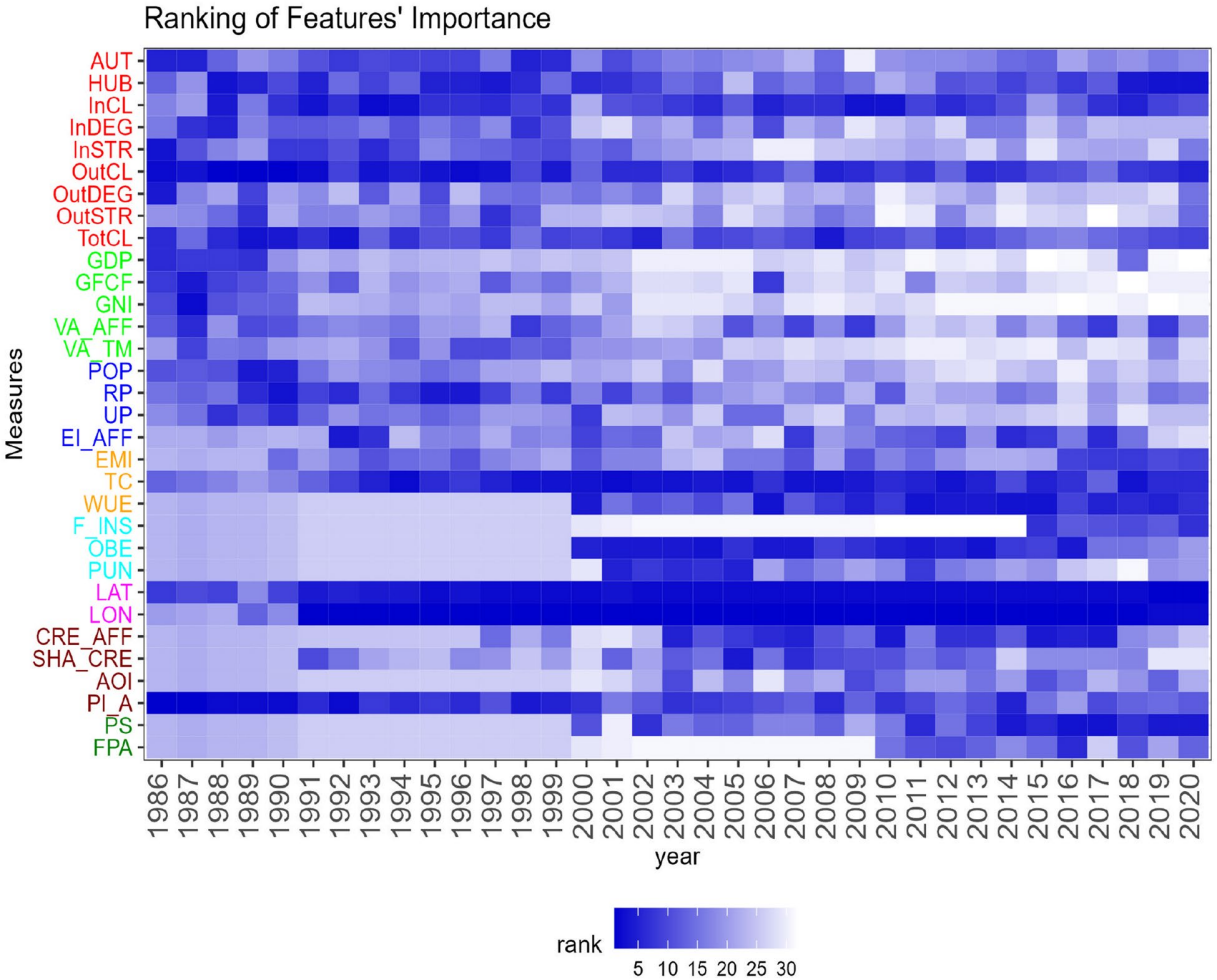
Ranking of features’ importance over time based on Mean Decrease Accuracy, using communities detected on the networks $G_{t}$. Features are represented by shades of blue, with darker blue indicating a higher rank. The *y*-axis labels has been grouped and colour-coded coloured according to macro-category. From top to bottom, the categories are: Topological Indicators, Macro Economic, Population and Employment, Climate and Sustainability, Food Security and Nutrition, Geographical, Investment and Production, Others. For more detailed information on the features and their categorization, refer to the Supplementary Material.

We focus now on the relevance of countries in the global agri-food trade network. To this end, we consider patterns of in- and out-strength in the network $G_{t}$ in 2020 (see Figs. 3 and 4, Section 1.2, Supplementary Material) and we display in Fig.  2 the evolution of top ten ranking of exporters and importers.

The USA, China and European countries appear as the key players in terms of both exported and imported values. Despite China being the largest agricultural producer overall, the USA stands out with the highest export values throughout the entire period. Surprisingly, the Netherlands takes, likely due to its significant role as a major European shipping hub. Brazil, Germany, France, China, Spain, Italy, Canada and Belgium follow suit. Among exporters, some industrial countries experienced a decline^65^ in their export shares between the beginning of the period and 2006-2007. Particularly noteworthy is the reduction in France’s market share, mainly due to a loss in country’s competitiveness^66^. Similarly, Great Britain faced a significant drop in its ranking over time and, its future behaviour may be also affected by the implications of the Brexit Agreement regarding agricultural exports^67^.

Regarding imports, as of 2020, China became leading importer of agricultural goods worldwide, followed by the United States and Germany. The patterns among importing countries are more heterogeneous, with some industrial and developing nations showing increasing shares in the market. Most relevant changes are observed in China and Japan. China, once a small importer, has significantly increased its imports reaching the top positions. Conversely, Japan, previously one of the world’s largest importers, has dropped to the seventh position.

The corresponding figures related to the network $G^{Q}_{t}$ have been provided in the Supplementary Material (see Figs. 5, 6 and 7, Section 1.2, Supplementary Material). We observe significant similarities between the two networks. For instance the rank correlation between the strengths (in or out) of $G_{t}$ and $G^{W}_{t}$ is always higher than 0.80 (see Fig. 8, Section 1.2, Supplementary Material for details). While it is expected to find a relevant relation between the monetary value and volume of trade, it is interesting to note that specific countries belong to the top 10 positions in terms of the volume of exports but have a lower position when the monetary value is considered. In particular, Argentina, Ukraine and Russia show a higher ranking in recent years when the volume in tonnes is considered. This aspect can be explained by the export profile of these countries. Argentina, Ukraine, and Russia are known for their significant production and export of agricultural commodities such as grains, oilseeds and other food products. These commodities are indeed exported in large volumes, leading to high values in tonnes. However, the lower ranking in terms of monetary value could be attributed to factors such as differences in prices and market dynamics. Various economic factors, such as exchange rates, the concentration of lower-priced commodities being exported, global supply and demand, competition, market conditions, and trade agreements, can influence the monetary value of these exports. These factors affect the final prices of the commodities in the international market, thereby impacting the overall monetary value of the trade.

### Communities

We now turn out attention to the communities detected over time using the InfoMap procedure on each network $G_{t}$. The composition pattern of the communities is displayed in Fig. 3. The evolution of the network topology in terms of density, trade connections and intensities (see Fig. 1b) has a significant impact on the number of communities detected. In the early 1980s, we observed a very low number of communities (from 2 to 4). However, with the increased network connectivity and diversification of trade partners since 1995, the number of communities expanded (from 9 to 11). The structure of the global network of food and agricultural trade has become more decentralised. In the past a few large trading hubs dominated the trade network. However, with the expansion of trade and the emergence of new players, the number of hubs has increased over time and the dominance of individual hubs weakened^64^. More countries are now connected to a larger number of trade partners, which can enhance the buffer capacity and resilience of the network. It is important to note that our interpretation aligns with the view that denser networks with higher average connectivity tend to exhibit higher resilience to attacks^68,69^. However, different perspectives exist in the food trade and production literature. Some studies suggest that more connected networks may be susceptible to target attacks^70,71^, while others find that the food production network is more interconnected, but not necessarily less stable^72^. The dynamics of the agri-food trade network and its implications on stability and resilience are complex and may vary under different circumstances.

According to the composition displayed in Fig. 3, three main communities are observed at the beginning of the period (1986-1990). The composition of these communities is influenced by the sharp increase in hostility between the United States and the Soviet Union during the last phase of the Cold War. The first community includes North and South America, Europe, a large part of Africa, East and South-East Asia and Oceania. The second community involves Russia, Soviet satellite countries (as Bulgaria, Czechoslovakia, Hungary, Poland and Romania) and countries with strong relations with Russia (like Cambodia, Vietnam and Cuba). A third residual community is formed by countries in the Middle East and North Africa (MENA). Despite the heterogeneity among these countries, relevant trade connections within MENA are present^73^. As highlighted by FAO^64^, regionalization of agri-food trade has increased since 1995, with a higher tendency of countries to trade more within a region than with countries outside the region. Countries tend to form specific communities and trade more within these groups, which may be regional or expand to include countries across regions. These patterns gradually reflect on the network over time and are often shaped by geographic proximity and economic integration fostered by trade agreements^27,28,74^. Our findings are in line with some previous literature based on older data^26,27^, but we also identify several additional specificities. Over the longer time period considered, North American countries and Mexico form a community together with China, Japan and Australia, confirming previous findings^26^. This result emphasises the relevance of connections between these countries at an aggregate level. However, works that disentangle products^27^ or focus on virtual water trade values^25^ suggest that China, Japan and Australia typically belong to different communities, orbiting around other communities such as the North American, South American and Russian ones. Our results show that Russia generally forms a second community together with Central Asian, Caucasian and East-European states. However this second community’s composition reduces over time and is limited only to Central Asian and Caucasian countries in 2020. A big, unified and independent European community emerges involving countries of the Maghreb region, in line with the EU agri-food trade’s solid growth in the last decade. We also observe other differences with respect to the literature^26^, with other strong communities that emerge. An enlarging community formed by MENA countries and India is noticeable. Relevant economies of South America, appear to be more connected with the US community and less with Europe. It is interesting to notice the increasing process of regionalization in Sub Saharian Africa, where, except for Nigeria, that is connected to American countries, two communities emerge orbiting around South Africa and Kenya, respectively. This result confirms findings that show that African countries have relatively good geographic diversification and do not seem to depend largely on a single country. Countries in sub-Saharan Africa are relatively less connected to other countries in the global network, due to significantly higher trade costs than high-income economies. But, at the same time, an increasing intra-regional trade can be observed^75^.

Additionally, we have applied the InfoMap procedure to the networks $G^{W}_{t}$ and the composition of communities is reported in Fig. 9, Section 2, Supplementary Material. At first glance, we notice significant overlapping between communities detected using monetary values and volume of trade. To assess the degree of similarity we have computed the Jaccard index^76,77^, based on the ratio between countries classified in the same way and the total number of nodes (see Fig. 10, Section 2, Supplementary Material for the details). We observe a level of similarity very close to 1 in the first period and an average value of roughly 70% over the whole period. When taking the volume of trades into consideration, a higher number of communities becomes apparent. However, these communities are frequently obtained as a decomposition of single communities observed in $G_{t}$. Typically, more robust subgroups can emerge. Significant disparities are evident during the early 21st century (from 2000 to 2010), where China, Australia, ASEAN countries and Japan form a distinct community compared to North America. During the same period, Argentina and Brazil, distinguished by their high out-degree and out-strength, form a distinct community, collaborating closely also through the Mercosur regional trade bloc. Likewise, India, Nepal, and often Pakistan exhibit separate behaviour from the Middle East, constituting their own distinct community.

### Characterizing communities: results and discussion

As previously described, we applied a random forest methodology to predict the community composition based on a set of features. For each node we considered 32 different attributes that encompass various facets of the agri-food trade dynamics (see Supplementary Material, Section 3.1 for a list and a description of the features used in the analysis). These attributes include topological indicators that assess the node’s relevance in the network in terms of centrality and clustering, geographical aspects such as latitude and longitude, macro-economic indices related to the overall economy and the agricultural sector, factors connected to investments and production, demographic indicators regarding population evolution and composition, sustainability indices concerning green-house gas emissions and water efficiency, meteorological aspects associated with climate change and elements related to food security and nutrition.

The model was initially trained using data from the entire time period, with a standard 70%-30% split between the training and test sets. To optimise the number of variables, we applied both a grid search algorithm and minimised the Out-of-bag error. Both methods yielded similar results in terms of number of variables and level of accuracy. The optimal choice was to use 19 variables, resulting in an accuracy of approximately 85%. After selecting the model’s parameters, we applied it separately for each time period. The model consistently achieved very high levels of accuracies even when applied separately for each year (see Fig. 11, Section 3.2, Supplementary Material for details). We also evaluated the importance of features using alternative methods, namely MDA, MDI and permutation feature. Since the rank correlation among these methods was very high, we report only the results based on the MDA approach in Fig.  4.

It is interesting to observe the significant change over time in the top variables that influence the formation of communities. In the initial periods, classical economic variables, such as GDP, Gross Fixed Capital Formation and Gross National Income, along with demographic and topological indicators, play a primary role. Consequently, communities emerge, formed by countries showing similarities in terms of population composition between rural and urban areas, macro-economic behaviour and their relevance as nodes in the network, in terms of interconnection and centrality.

Subsequently, confirming previous findings^28^, geographical aspects, such as latitude and longitude, emerge as key factors along with the country’s role in the trade network. This is consistent with the growth in food and agricultural trade in the new millennium, which has been accompanied by increased connectivity between countries. More countries have expanded their participation in global food trade, resulting in a changing landscape and geography of trade. The specific patterns of trades between countries create a network of trade that reflects each country’s relative position and important features of the global market. Countries with many trade partners and high trade intensity are located closer to the core of this trade network, while those with few trade partners and low trade intensity are situated at the periphery.

Other noteworthy aspects are the increasing role of the climate change and sustainability and food nutrition elements over time. The relevance of temperature change is highlighted, emphasizing its critical impact on agri-food trade^79^. There is indeed a strong connection between temperature change and trade, as changes in temperature can have significant impacts on agricultural production and trade patterns. Rising temperatures can lead to droughts, floods, and other extreme weather events, which can negatively impact crop yields and livestock production. This, in turn, can lead to lower food production, higher food prices, and changes in the types of crops grown in different regions. As a result, temperature change significantly impacts agri-food trade, as countries may need to import more food to compensate for domestic production shortfalls, or adapt their trade patterns to source different types of food from different regions. Regarding sustainability factors, water use efficiency (WUE) shows an increasing importance in our results. For example, as pointed out in the literature^29^, trade agreements favour goods with higher water productivity. Differences between trade links covered by agreements and those not covered are amplified when focusing on blue water. This suggests that countries linked by trade agreements can allocate irrigation water more efficiently by directing it towards more productive crops, both nutritionally and economically.

Also the increasing attention to food security and nutrition has been significant in recent years, influencing the composition of communities. Previous studies have analysed the relationship between participation in global food trade and the prevalence of overweight and obesity, showing that increased competitiveness in food supply leads to higher diversity and quality of available food at national level^80,81^. Additionally, the role of political stability has grown over time, in line with current literature showing that similarities in political attributes and institutional factors, such as economic freedom and government governance, significantly impact bilateral trade of food products^14,28,43^.

The main behaviours described above are confirmed also when focusing on the trade volume in tonnes (see Fig. 12, Section 3.2, Supplementary Material for accuracy and Fig. 13, Section 3.2, Supplementary Material for the ranking of feature importance). The degree of similarity between the rankings of features obtained on the two networks based on monetary values and quantities is measured using cosine similarity^82^ for each feature and for each year (see Fig. 14, Section 3.2, Supplementary Material for the similarity plots). We notice a similarity between 87 and 97% over time, confirming that results obtained using monetary values also provide a good representation of the trade volumes. However, it is noteworthy to examine the similarity at the feature level. Specifically, we observe a lower similarity for weighted topological indicators, prevalence of undernourishment (PUN) and the gross production index in agriculture (PI_A). Weighted topological indicators exhibit a greater importance of out-strength and clustering when the volume in tonnes is considered. This is partially related to the emergence of countries such as Argentina, Russia and Ukraine, which have higher rankings when considering trade volumes. The production index shows instead on average a higher relevance when monetary values are considered, suggesting a stronger impact on the formation of communities due to its relation with price behaviour and stability. Regarding the prevalence of undernourishment (PUN), this variable appears significant only for a shorter period when monetary values are considered, particularly during the years when an improvement in the value of this index was observed globally^83,84^.

Next, we focus on the most relevant features and evaluate their possibility of over-expression in specific communities obtained using the networks $G_{t}$ and $G^{W}_{t}$. Main results in terms of variables and classes that resulted significant for each community in the two networks over time have been displayed in Figs. 15 and 16, Section 3.2, Supplementary Material. In the early period analysed (1986), , the procedure does not provide other relevant insights due to the emergence of very large communities that include countries with diverse characteristics.Community 1 is particularly large and includes heterogeneous countries, but it shows the highest average values in terms of topological indicators. Community 2, which includes countries that were part of the Soviet Union and Eastern Europe, exhibits the highest average values in terms of population and economic indicators related to agriculture. Community 3, involving Middle Eastern countries, emphasises the relevance of latitude and longitude, being located in a well-defined area between 24th and 33rd parallel north and between 30th and 71st meridian.

In contrast, in more recent years, there is higher homogeneity in terms of features. As seen in previous literature^26^, all communities in both networks are characterised by an over-expression of specific values of latitude and longitude, confirming the importance of geographical relationship in agri-food trade (see Tables 3 and 4, Section 3.2, Supplementary Material for a list of relevant variables in 2020).

By extending the analysis to other attributes and focusing on the year 2020 for brevity, we can identify the main drivers characterising the detected communities. In both networks, community one, including America, China, Japan and Oceania, is characterised by a very high proportion of countries playing a key role in the network. These countries show high export values (out-strength), high levels of interconnection (clustering) and rank among the top countries in terms of directed centrality scores (both hub and authority). Additionally, the Value Added in Agriculture, Forestry, and Fishing (VA_AFF) for these countries, measured in US dollars, is remarkably high on average. Notably, seven out of the top ten countries worldwide in terms of VA_AFF belongs to community 1.

Topological indices are also key drivers for community 4, which involves European countries. This community stands out with a higher average degree due to the number of in- and out-trade relationships. Prominent countries within this community include Belgium, Netherlands, France, Germany, UK, Italy, and Spain, ranking within the top 15 in terms of in- and out-degree. Furthermore, the community’s composition is driven by factors such as a robust manufacturing sector and a well-developed financial market, with significant credit allocation to the agricultural sector. These characteristics further define and distinguish this particular community. This community also shows higher values of temperature change^85^ (TC), which is a distinguishing characteristic in both community 2 (including Russia and neighbouring countries) and community 4. Additionally, as stressed above, low levels of political stability (PS) represents another driver for the composition of food community. Political instability can significantly impact food trade, leading to disruptions in the movement of agricultural products across borders and creating uncertainties for importers and exporters alike. It can hinder the smooth flow of food commodities, potentially leading to food shortages, price volatility, and economic challenges for nations heavily reliant on international food trade. This driver, together with high levels of prevalence of severe food insecurity in the total population appear crucial factors also for community three, to which belongs Middle Eastern countries. In contrast to other communities, community 6, which consists of Latin American and Caribbean countries, exhibits a significant lower average value added related to Agriculture, Forestry, and Fishing as a percentage of GDP. This trend aligns with the observed reduction in this percentage, relative to GDP, over time, which can be attributed to challenges posed by climate-related factors such as extreme weather events and droughts, impacting agricultural production and output in these nations^64^.

Furthermore, these countries display limited diversification of their trade partners, resulting in relatively low values of in and out trade connections.

## Conclusions

The proposed approach leverages network theory to investigate the composition of communities of countries in agri-food trade over time. In line with the existing literature, the analysis reveals significant changes in the topological structure of the network, with an increase in the number of trade partners and higher trade intensity over the years. By employing a random forest approach and evaluating features’ over-expression, we identify key attributes that characterise the community composition. Geographical connections remain a primary driver of agri-food trade, with many countries still relying on regional or local suppliers for certain products. Topological indicators and the centrality of countries in the network play a crucial role in shaping larger communities, as nodes with comparable importance or influence tend to cluster together.

Additionally, the results highlight the growing significance of climate and sustainability issues in recent years. The composition of countries, with vastly different patterns worldwide, can also be partially attributed to the crucial aspects of food nutrition and food security. These factors play a significant role in understanding the variations observed among nations. Political stability further contributes to the composition of communities, in line with previous findings^43^, underscoring the impact of institutional factors on bilateral food product trade.

Therefore, several points have to be considered to fulfil the vision of FAO’s vision of a world free from hunger and malnutrition. These factors include population growth, dietary choices, technological progress, income distribution, natural resource use, climate change, and conflict resolution efforts. Policymakers need to design and implement trade policies and practices that support healthy and sustainable food systems while promoting access to affordable, nutritious, and culturally appropriate foods for all. Such measures may involve promoting more sustainable food production and trade practices, improving nutrition education and awareness, fostering equitable and resilient trade relationships, and addressing climate change through climate-friendly regulations and international cooperation.

In this context, the identification of main drivers of trade relationships can represent a suitable tool for the development of policies that are, at the same time, consistent with international trade rules and does not unfairly disadvantage developing countries. Future research in this area could explore commodity-level analysis, which can provide valuable insights into understanding alternative trade relationships and the underlying factors. By combining the proposed methodology for identifying relevant features with a multi-network approach^27,86^, researchers could highlight key drivers for each commodity, enabling the development of more targeted and effective policies consistent with international trade rules and beneficial for developing countries.

### Supplementary Information

Supplementary Information.

## Data Availability

All datasets used in this study are publicly available and have been sourced from FAOSTAT^32^. The datasets can be accessed at the following link: https://www.fao.org/faostat/en/#data.
