# Supplementary material for: Unraveling the key drivers of community composition in the agri-food trade network
Source: Sci Rep. 2023 Aug 26;13:13966. doi: 10.1038/s41598-023-41038-z (PMC10460445; doi:10.1038/s41598-023-41038-z)
Supplement: Supplementary file 1 — Supplementary Information. [file 41598_2023_41038_MOESM1_ESM.pdf]

Supplementary Material
Unraveling the Key Drivers of Community Composition in the Agri-food Trade Network

Gian Paolo Clemente[1,*], Alessandra Cornaro[2,+], and Francesco Della Corte[1,+]

[1]Università Cattolica del Sacro Cuore, Milan, Italy
[2]Università degli Studi di Milano - Bicocca, Milan, Italy
[*]gianpaolo.clemente@unicatt.it
[+]alessandra.cornaro@unimib.it, francesco.dellacorte1@unicatt.it

ABSTRACT

In the complex global food system, the dynamics associated with international food trade have become crucial determinants of food security. In this paper, we employ a community detection approach along with a supervised learning technique to explore the evolution of communities in the agri-food trade network and to identify key factors influencing their composition. By leveraging a large dataset that includes both volume and monetary value of trades, we identify similarities between countries and uncover the primary drivers that shape trade dynamics over time. The analysis also takes into account the impact of evolving climate conditions on food production and trading. The results highlight how the network's topological structure is continuously evolving, influencing the composition of communities over time. Alongside geographical proximity and geo-political relations, our analysis identifies sustainability, climate and food nutrition aspects as emerging factors that contribute to explaining trade relationships. These findings shed light on the intricate interactions within the global food trade system and provide valuable insights into the factors affecting its stability.

1 Supplementary Material for Section Preliminaries

1.1 List of Countries

Table 1 presents the list of countries and their corresponding codes used in the analysis.

Country	Code	Country	Code
Afghanistan	AFG	Albania	ALB
Algeria	DZA	Angola	AGO
Antigua and Barbuda	ATG	Argentina	ARG
Armenia	ARM	Australia	AUS
Austria	AUT	Azerbaijan	AZE
Bahamas	BHS	Bahrain	BHR
Bangladesh	BGD	Barbados	BRB
Belarus	BLR	Belgium	BEL
Belize	BLZ	Benin	BEN
Bhutan	BTN	Bolivia (Plurinational State of)	BOL
Bosnia and Herzegovina	BIH	Botswana	BWA
Brazil	BRA	Brunei Darussalam	BRN
Bulgaria	BGR	Burkina Faso	BFA
Burundi	BDI	Cabo Verde	CPV
Cambodia	KHM	Cameroon	CMR
Canada	CAN	Central African Republic	CAF
Chad	TCD	Chile	CHL
China, Hong Kong SAR	HKG	China, Macao SAR	MAC
China	CHN	China, Taiwan Province of	TWN
Colombia	COL	Comoros	COM
Congo	COG	Cook Islands	COK
Costa Rica	CRI	Cote d'Ivoire	CIV
Croatia	HRV	Cuba	CUB
Cyprus	CYP	Czechia	CZE
Democratic People's Republic of Korea	PRK	Democratic Republic of the Congo	COD
Denmark	DNK	Libya	LBY
Djibouti	DJI	Dominica	DMA
Dominican Republic	DOM	Ecuador	ECU
Egypt	EGY	El Salvador	SLV
Equatorial Guinea	GNQ	Eritrea	ERI
Estonia	EST	Eswatini	SWZ
Ethiopia	ETH	Faroe Islands	FRO
Fiji	FJI	Finland	FIN
France	FRA	French Polynesia	PYF
Gabon	GAB	Gambia	GMB
Georgia	GEO	Germany	DEU
Ghana	GHA	Greece	GRC
Grenada	GRD	Guatemala	GTM
Guinea	GIN	Guinea-Bissau	GNB
Guyana	GUY	Haiti	HTI
Honduras	HND	Hungary	HUN
Iceland	ISL	India	IND
Indonesia	IDN	Iran (Islamic Republic of)	IRN
Iraq	IRQ	Ireland	IRL
Israel	ISR	Italy	ITA
Jamaica	JAM	Japan	JPN
Jordan	JOR	Kazakhstan	KAZ
Kenya	KEN	Kiribati	KIR
Kuwait	KWT	Kyrgyzstan	KGZ
Lao People's Democratic Republic	LAO	Latvia	LVA

Country	Code	Country	Code
Lebanon	LBN	Lesotho	LSO
Liberia	LBR	Lithuania	LTU
Luxembourg	LUX	Madagascar	MDG
Malawi	MWI	Malaysia	MYS
Maldives	MDV	Mali	MLI
Malta	MLT	Marshall Islands	MHL
Mauritania	MRT	Mauritius	MUS
Mexico	MEX	Micronesia (Federated States of)	FSM
Mongolia	MNG	Montenegro	MNE
Morocco	MAR	Mozambique	MOZ
Myanmar	MMR	Namibia	NAM
Nauru	NRU	Nepal	NPL
Netherlands	NLD	New Caledonia	NCL
New Zealand	NZL	Nicaragua	NIC
Niger	NER	Nigeria	NGA
Niue	NIU	North Macedonia	MKD
Norway	NOR	Oman	OMN
Pakistan	PAK	Palestine	PSE
Panama	PAN	Papua New Guinea	PNG
Paraguay	PRY	Peru	PER
Philippines	PHL	Poland	POL
Portugal	PRT	Qatar	QAT
Republic of Korea	KOR	Republic of Moldova	MDA
Romania	ROU	Russia	RUS
Rwanda	RWA	Saint Kitts and Nevis	KNA
Saint Lucia	LCA	Saint Vincent and the Grenadines	VCT
Samoa	WSM	Sao Tome and Principe	STP
Saudi Arabia	SAU	Senegal	SEN
Serbia	SRB	Seychelles	SYC
Sierra Leone	SLE	Singapore	SGP
Slovakia	SVK	Slovenia	SVN
Solomon Islands	SLB	Somalia	SOM
South Africa	ZAF	South Sudan	SSD
Spain	ESP	Sri Lanka	LKA
Sudan	SDN	Suriname	SUR
Sweden	SWE	Switzerland	CHE
Syrian Arab Republic	SYR	Tajikistan	TJK
Thailand	THA	Timor-Leste	TLS
Togo	TGO	Tokelau	TKL
Tonga	TON	Trinidad and Tobago	TTO
Tunisia	TUN	Turkey	TUR
Turkmenistan	TKM	Tuvalu	TUV
Uganda	UGA	Ukraine	UKR
United Arab Emirates	ARE	United Kingdom of Great Britain and Northern Ireland	GBR
United Republic of Tanzania	TZA	United States of America	USA
Uruguay	URY	Uzbekistan	UZB
Vanuatu	VUT	Venezuela (Bolivarian Republic of)	VEN
Viet Nam	VNM	Yemen	YEM
Zambia	ZMB	Zimbabwe	ZWE

Table 1. List of countries and ISO codes

1.2 Additional Figures and Results related to Section Preliminaries

Here, we present additional figures and results that support the comments provided in the *Preliminaries* section of the main paper.

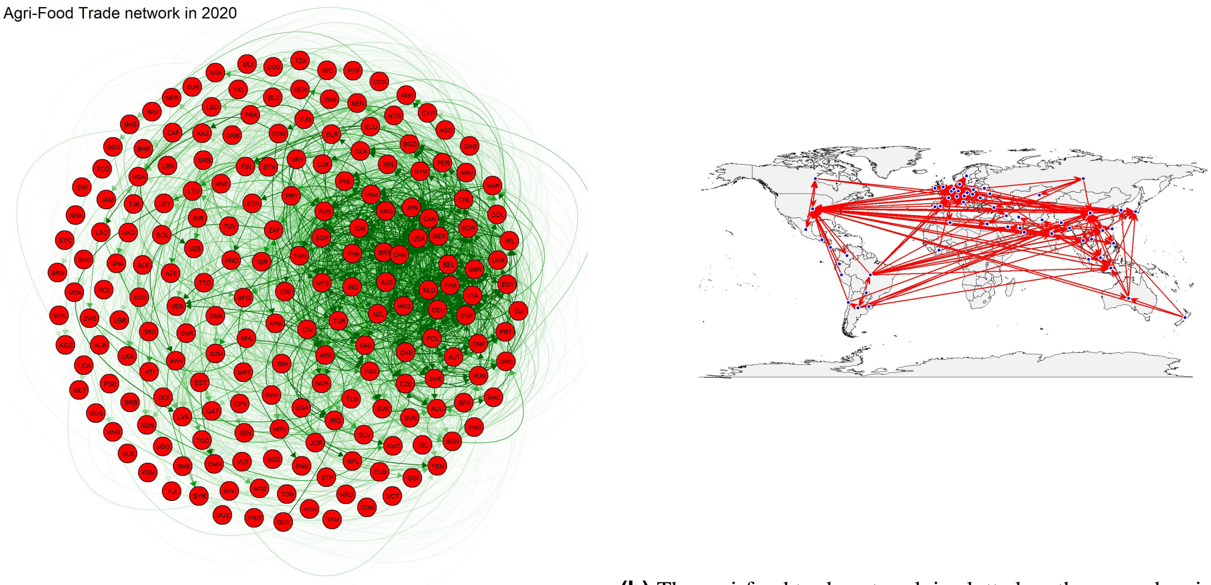

(a) Agri-food trade network

(b) The agri-food trade network is plotted on the map, showing only edges with weights higher than the 99th quantile of the weights' distribution. The map was generated using R software[1].

Figure 1. Agri-food trade network G_t, that represents the trade relationships in 2020, with edge weights based on the amounts of import and export measured in thousands of US dollars.

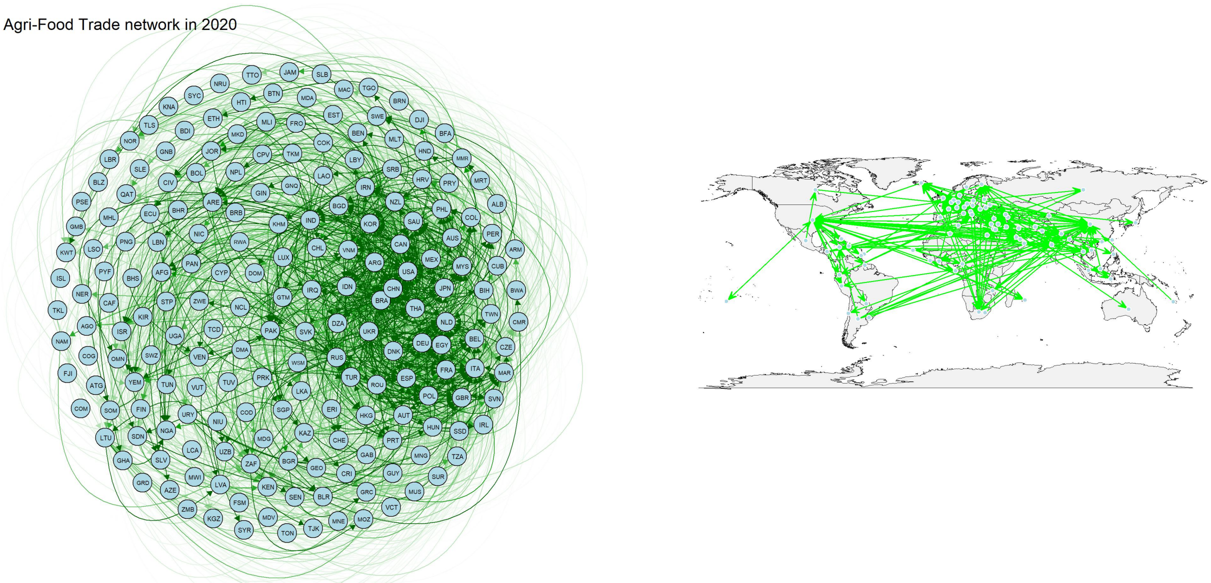

(a) Agri-food trade network

(b) The agri-food trade network is plotted on the map, showing only edges with weights higher than the 99th quantile of the weights' distribution. The map was generated using R software[1].

Figure 2. Agri-food trade network G_t^Q, that represents the trade relationships in 2020, with edge weights based on the quantities of import and export measured in tonnes.

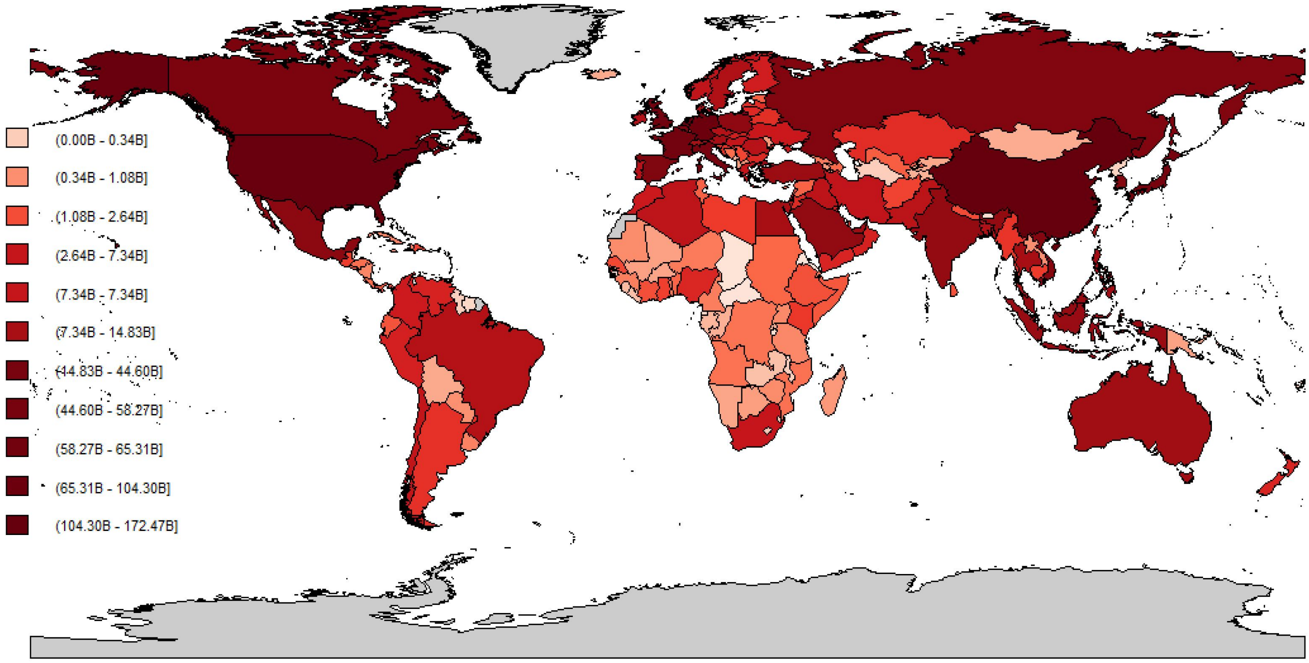

Figure 3. The in-strength of the agri-food trade network in 2020 is represented in billions of US dollars, with darker red colors indicating higher values. The map was generated using R software[1].

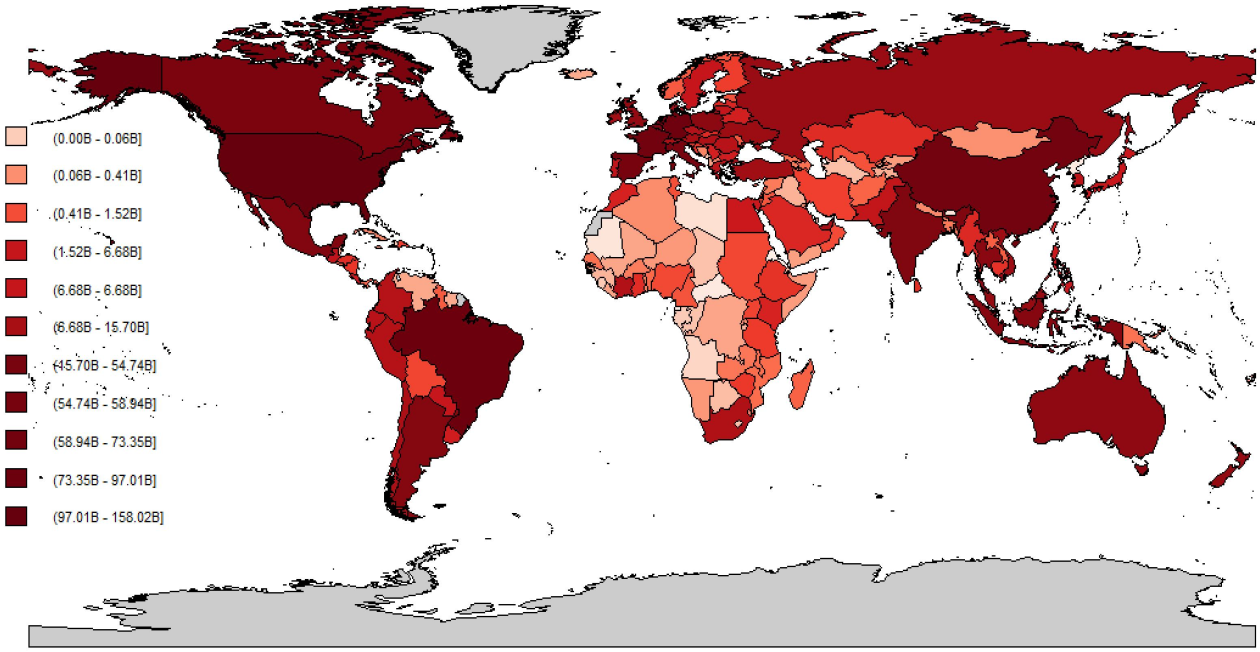

Figure 4. The out-strength of the agri-food trade network in 2020 is represented in billions of US dollars, with darker red colors indicating higher values. The map was generated using R software[1].

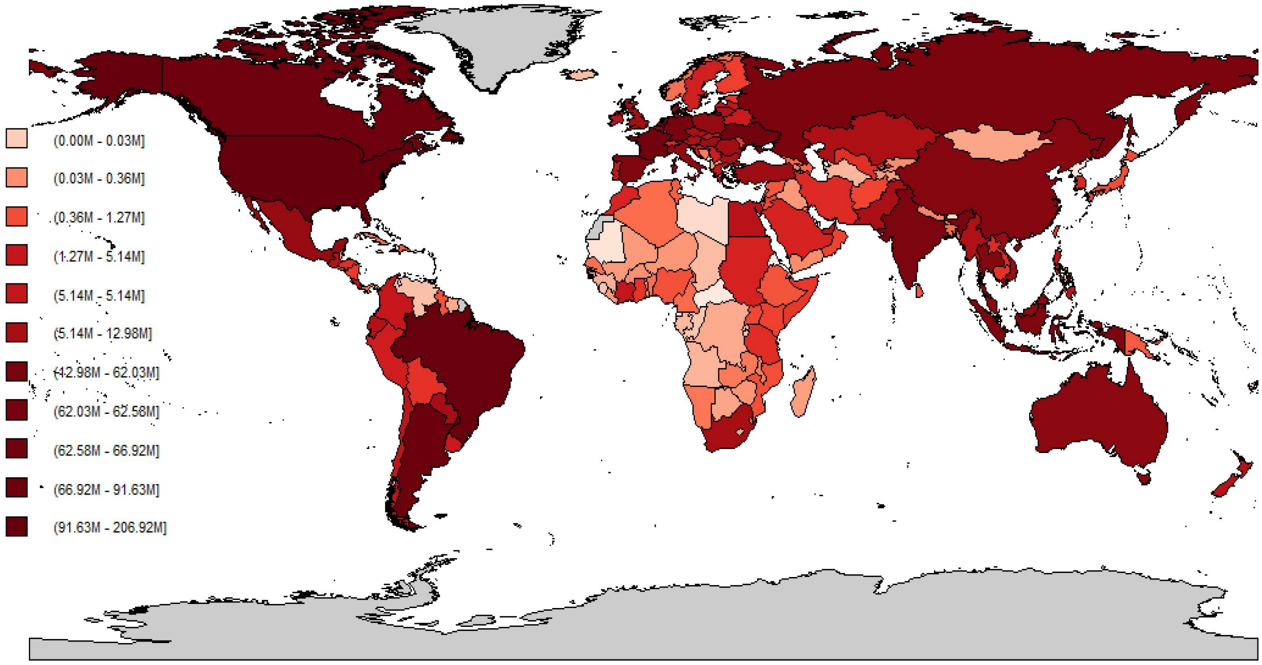

Figure 5. The in-strength of the agri-food trade network in 2020 is expressed in millions of tonnes, with darker red indicating higher values. The map was generated using R software[1].

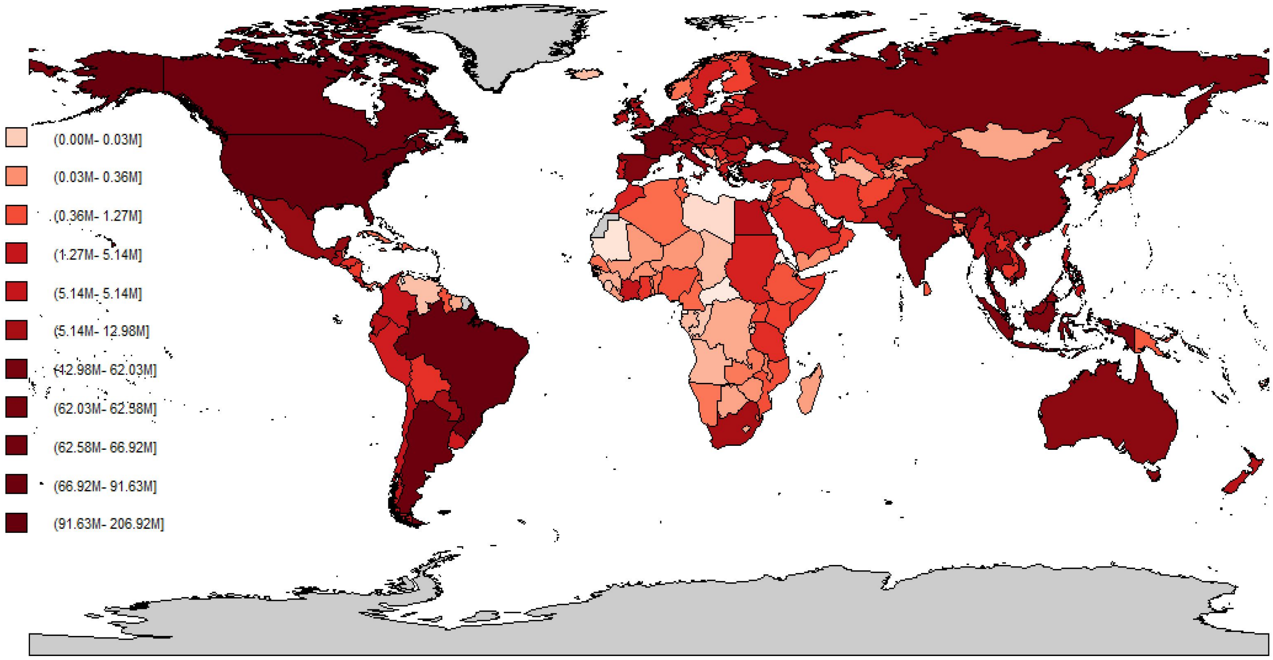

Figure 6. The out-strength of the agri-food trade network in 2020 is expressed in millions of tonnes, with darker red indicating higher values. The map was generated using R software[1].

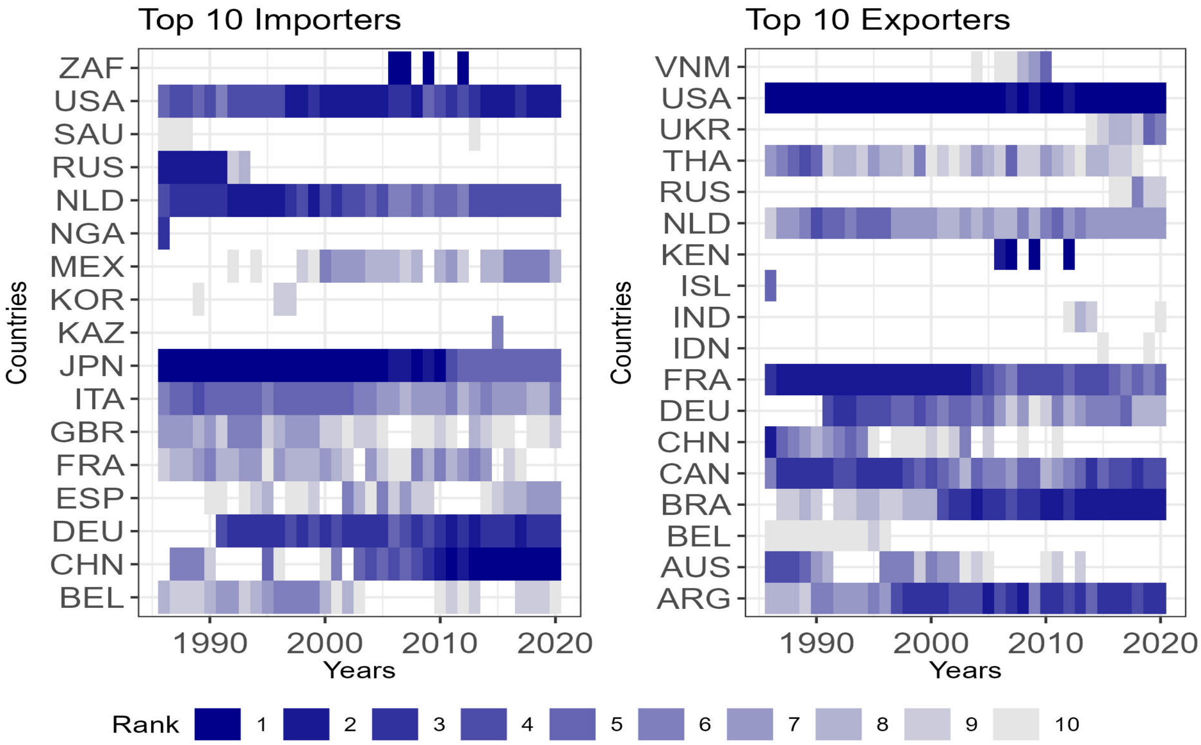

Figure 7. Evolution of the rankings of the top ten exporters and importers in terms of tonnes over time. We only show countries that were among the top ten in at least one of the analysed years. If a country had a lower rank in a specific year, an empty value is displayed.

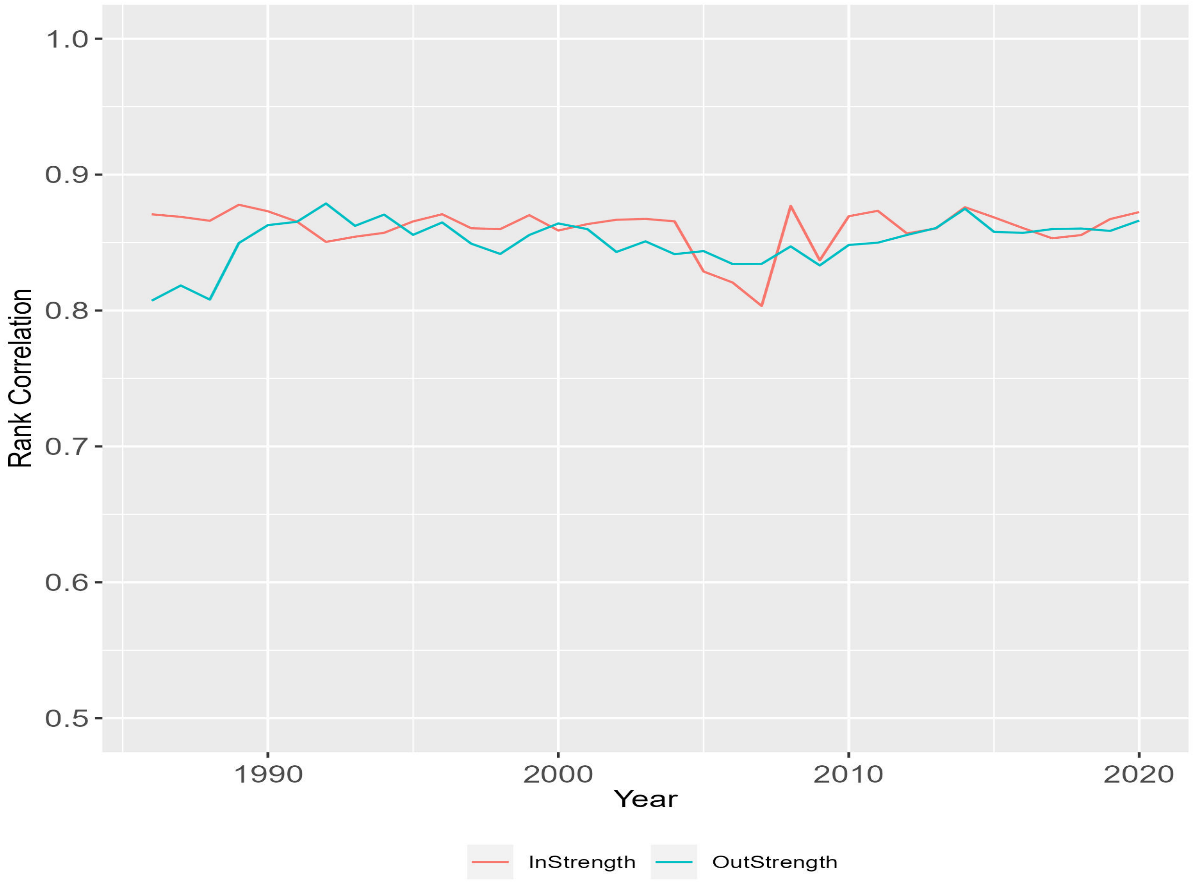

Figure 8. The Kendall rank correlation between in-strength values based on networks G_t and G_t^W is shown in red, while the rank correlation based on out-strength values is displayed in cyan.

2 Additional figures and results related to Section Communities

In this section, we present additional figures and results that support the comments provided in the *Communities* section of the main paper.

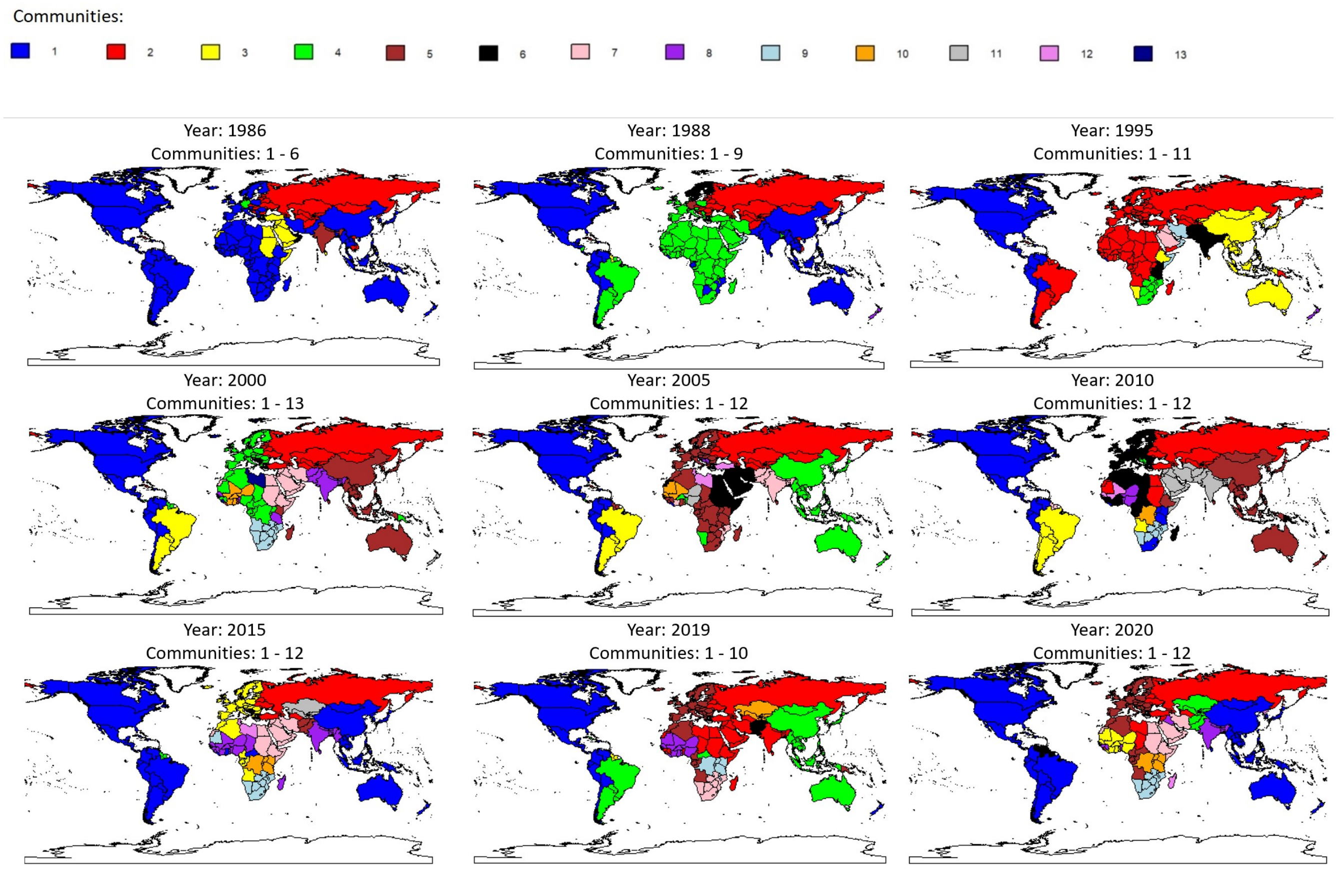

Figure 9. Communities detected via the InfoMap approach based on networks G_t^W where the weights are related to the quantity of imports and exports (in tonnes). The number of communities detected in each year is displayed above each figure. The maps were generated using R software[1].

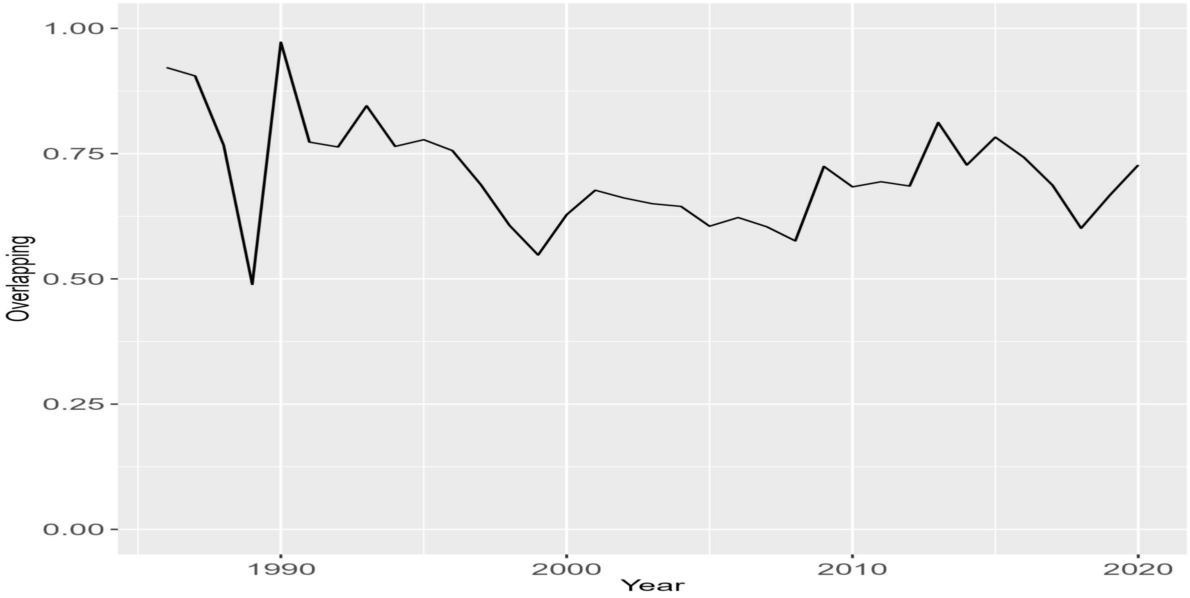

Figure 10. Degree of overlapping based on the Jaccard index between communities detected via the InfoMap approach in two networks: one with weights related to the quantity of imports and exports (in tonnes), and the other with weights related to the monetary value of imports and exports (in thousands of US dollars).

3 Additional aspects related to the Section: Characterizing Communities: results and discussion

3.1 Description and list of features

We provide a description of the features used in the random forest method and for characterizing communities. Below is a summary list and brief description of the variables used as node attributes (also refer to Table 2).

- Topological indicators
  - *Node in-degree*[2]. It is the number of incoming edges that are directed towards a specific node in a network, often used as a simple measure of centrality of a node within a network.
  - *Node out-degree*[2]. It is the number of outgoing edges from a specific node in a network.
  - *Node in-strength*[2]. It is the sum of the weights of all incoming edges directed towards a specific node in a network, measuring the measures total in-flows of a node.
  - *Node out-strength*[2]. It is the sum of the weights of all outgoing edges from a specific node in a network, measuring the total out-flows of a node.
  - *Local directed and weighted total clustering*[3,4]. Clustering in directed networks is a measure of the extent to which nodes in a network tend to form groups or clusters of interconnected nodes with arcs. It is measured by calculating the fraction of all possible directed triangles, properly weighted, that are present in the network. Clustering in directed networks can help identify patterns of connectivity and level of interconnections within the network.
  - *Local directed and weighted in-clustering*[3,4]. It considers only "in" triangles, i.e. triangles in which the neighbours of a node i point to the node and they are also connected each other.
  - *Directed and weighted out-clustering*[3,4]. It considers only out-triangles, i.e. triangles in which two neighbours of a node i are connected by an incoming arc from the node i and they are also connected each other.
  - *Hub centrality score*[5]: The hub centrality score is based on the idea that important nodes in a network should have many connections to other nodes that are themselves well-connected. Nodes with high hub centrality scores are those that are connected to many nodes with high authority centrality scores, suggesting that they act as important hubs or connectors in the network.

- *Authority centrality score*[5]. The authority centrality score identifies nodes that are highly connected to by nodes with high hub centrality scores. In other words, nodes with high authority centrality scores are those that are frequently pointed to by other well-connected nodes in the network.

- Macro-Economic indicators based on FAO data[6]

  - *Gross Domestic Product (GDP)* (US dollars). Total value of all goods and services produced within a country's borders during a given period. It includes all final goods and services produced for consumption, investment, government, and exports, and is used to measure the size and growth of an economy.

  - *Gross Fixed Capital Formation* (US dollars). Total investment in fixed assets, such as machinery, buildings, infrastructure, and equipment. It is an important indicator of the level of investment in an economy, as it reflects the total amount of resources that are being allocated towards the production of capital goods, which are essential for increasing the productivity and capacity of an economy in the long run.

  - *Gross National Income* (GNI) (US dollars). Total income generated by a country's residents, domestically and abroad. It is calculated as the sum of all incomes earned by a country's residents, including wages, salaries, profits, and property income, minus any taxes and subsidies paid to foreign entities. Therefore, it measures the total value of all goods and services produced within a country's borders. However, while GDP only includes income generated within a country's borders, GNI includes income earned by its residents abroad, such as profits from foreign investments or remittances sent by workers living abroad.

  - *Global Agriculture, Forest and Fishing Value Added* (US dollars). Total economic value generated by these three primary industries in a country. It represents the contribution of these sectors to the overall GDP and it is calculated by subtracting the cost of intermediate inputs from the total output.

  - *Global Total Manufacturing Value Added* (US dollars). Value added of the manufacturing industries.

- Population and employment indicators based on FAO data[6]

  - *Total Population* (thousands). United Nations population estimates based on World Population Prospects.

  - *Rural Population* (thousands). It refers to the population that resides in rural areas, which are defined as areas outside of urban centres with a lower population density and a lower degree of economic and social development. Rural areas can include farmland, forests, small towns, and villages. Data are based on World Urbanization Prospects of the United Nations.

  - *Urban Population* (thousands). It refers to the population that resides in urban areas, which are defined as areas with a high population density and a high degree of economic and social development. Urban areas can include cities, towns, and other densely populated settlements. Data are based on World Urbanization Prospects of the United Nations.

  - *Share of employment in agriculture, forestry and fishing in total employment* (percent). Proportion of people employed in these sectors compared to total employment.

- Climate and Sustainability indicators based on FAO data[6]

  - *Emissions* (kilotonnes). The domain includes methane (CH4) nitrous oxide (N2O) and carbon dioxide (CO2) emissions. Data have been collected following the Tier 1 methods of the Intergovernmental Panel on Climate Change (IPCC) Guidelines for National greenhouse gas (GHG) Inventories

  - *Mean surface temperature change* (Degree Celsius). Data are based on the publicly available GISTEMP data, the Global Surface Temperature Change data distributed by the National Aeronautics and Space Administration Goddard Institute for Space Studies.

  - *Water Use Efficiency* (US dollar per cubic meter). It is a measure of the economic value generated per unit volume of water used. It is calculated by dividing the total economic output generated by a particular sector or activity by the total volume of water used for that activity. Water use efficiency is an important indicator of sustainable water management, as it can help identify opportunities to increase the economic value generated from a given volume of water, and to reduce water use where possible.

- Food security and nutrition based on FAO data[6]

  - *Prevalence of severe food insecurity in the total population* (percent). The prevalence of severe food insecurity in the total population refers to the percentage of people in a given population who experience severe food insecurity. Severe food insecurity is defined as a condition where people lack access to sufficient quantities of food or are unable to acquire food in socially acceptable ways, which results in a significant risk of hunger, malnutrition, or other adverse health outcomes. It belongs to the Suite of Food Security Indicators defined by the Committee on World Food Security Round Table on hunger measurement.

  - *Prevalence of obesity in the adult population* (percent). It refers to the percentage of adults (18 years old and older) in a given population who have a body mass index of 30 or higher, indicating that they are severely overweight and at increased risk for a variety of health problems.

  - *Prevalence of undernourishment* (percent). It is a measure of the percentage of the population in a given area that is unable to acquire enough food to meet their daily energy requirements. It is defined as the percentage of the population whose dietary energy intake is below the minimum level needed to maintain an adequate level of physical activity and a healthy body weight. It belongs to the Suite of Food Security Indicators defined by the Committee on World Food Security Round Table on hunger measurement

- Geographic coordinates

  - *Latitude*

  - *Longitude*

- Investments and Production based on FAO data[6]

  - *Credit to Agriculture, Forestry and Fishing* (US dollars). It is based on the Credit to Agriculture dataset that considers national data on the amount of loans provided by the private/commercial banking sector to producers in agriculture, forestry and fishing, including household producers, cooperatives, and agro-businesses.

  - *Share of Credit to Agriculture, Forestry and Fishing* (percent). It considers the share of credit to agricultural producers with respect to the total credit.

  - *Agriculture orientation index* (AOI). The AOI for credit normalises the Credit to Agriculture, Forest and Fishing by dividing it by the share of agriculture in GDP. As such, it can provide a more accurate indication of the relative importance that banking sector places on financing the agricultural sector. An AOI lower than one indicates that the agriculture sector receives a credit share lower than its contribution to the economy, while an AOI greater than one indicates a credit share to the agriculture sector greater than its economic contribution.

  - *Gross Production Index in Agriculture*. It is an economic indicator that measures the level of production of agricultural goods within an economy. It is a subset of the overall Gross Production Index that measures the level of production of all goods and services within an economy.

- Other indicators based on FAO data[6]

  - *Political stability and absence of violence and terrorism*. It is an index that measures perceptions of the likelihood that the government will be destabilised or overthrown by unconstitutional or violent means, including politically-motivated violence and terrorism.

  - *Indicator of Food Price Anomalies*. It is an economic indicator used to identify unusual or extreme changes in food prices. It is calculated by comparing the current Consumer Food Price Index, which measures the average change in prices of food products consumed by households, to its historical average or trend. If the current index value is significantly above or below the historical average, this suggests the occurrence of a food price anomaly.

Type	Attribute	Code
Investments and Production	Agriculture orientation index	AOI
Topological indicator	Authority centrality score	AUT
Investments and Production	Credit to Agriculture, Forestry and Fishing	CRE_AFF
Population and employment	Share of employment in agriculture, forestry and fishing in total employment	EI_AFF
Climate and Sustainability	Emissions	EMI
Food security and nutrition	Prevalence of severe food insecurity in the total population	F_INS
Others	Indicator of Food Price Anomalies	FPA
Macro-economic indicator	Gross Domestic Product	GDP
Macro-economic indicator	Gross Fixed Capital Formation	GFCF
Macro-economic indicator	Gross National Income	GNI
Topological indicator	Hub centrality score	HUB
Topological indicator	Local Directed and weighted in-clustering	InCL
Topological indicator	Node in-degree	InDEG
Topological indicator	Node in-strength	InSTR
Geographic coordinates	Latitude	LAT
Geographic coordinates	Longitude	LON
Food security and nutrition	Prevalence of obesity in the adult population	OBE
Topological indicator	Local Directed and weighted out-clustering	OutCL
Topological indicator	Node out-degree	OutDEG
Topological indicator	Node out-strength	OutSTR
Investments and Production	Gross Production Index in Agriculture	PI_A
Population and employment	Total Population	POP
Others	Political stability and absence of violence and terrorism	PS
Food security and nutrition	Prevalence of undernourishment	PUN
Population and employment	Rural Population	RP
Investments and Production	Share of Credit to Agriculture, Forestry and Fishing	SHA_CRE
Climate and Sustainability	Mean surface temperature change	TC
Topological indicator	Local Directed and weighted total clustering	TotCL
Population and employment	Urban Population	UP
Macro-economic indicator	Global Agriculture, Forest and Fishing Value Added	VA_AFF
Macro-economic indicator	Global Total Manufacturing Value Added	VA_TM
Climate and Sustainability	Water Use Efficiency	WUE

Table 2. List of attributes and codes used in the related Figures

3.2 Additional Figures and Results

In this section, we present additional figures and tables that provide support for the comments made in the *Characterizing Communities: Results and Discussion* section of the main paper.

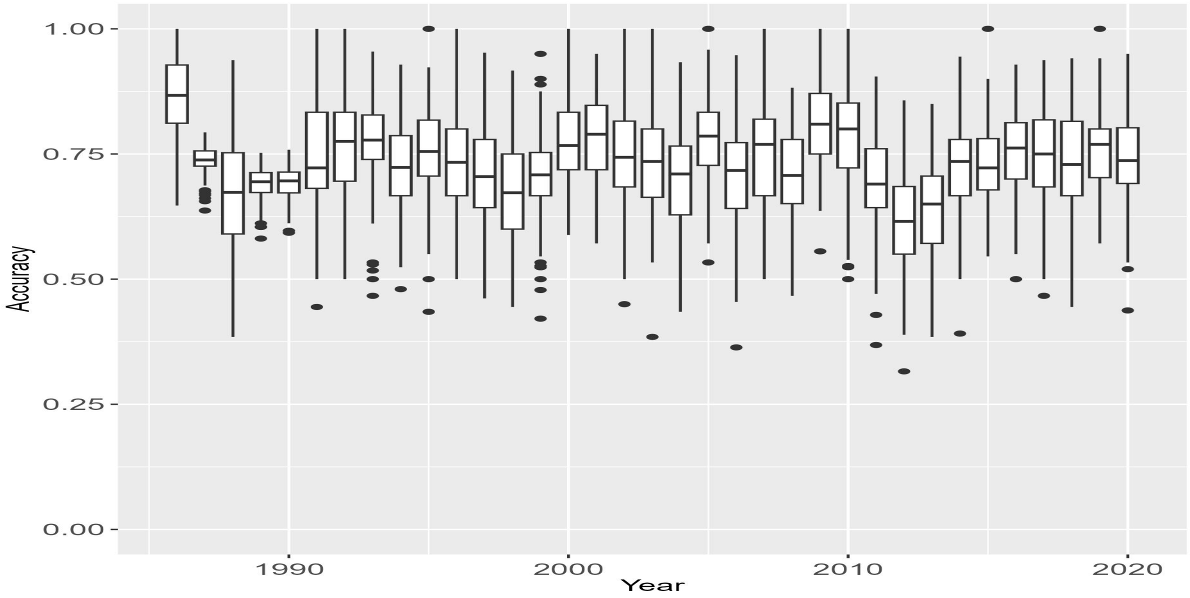

Figure 11. The boxplot illustrates the accuracy of 100 iterations of the random forest model for each year. The random forest model was applied to the communities detected by the InfoMap procedure on the network G_t. In each iteration, we sampled the training dataset and measured the accuracy on the test set.

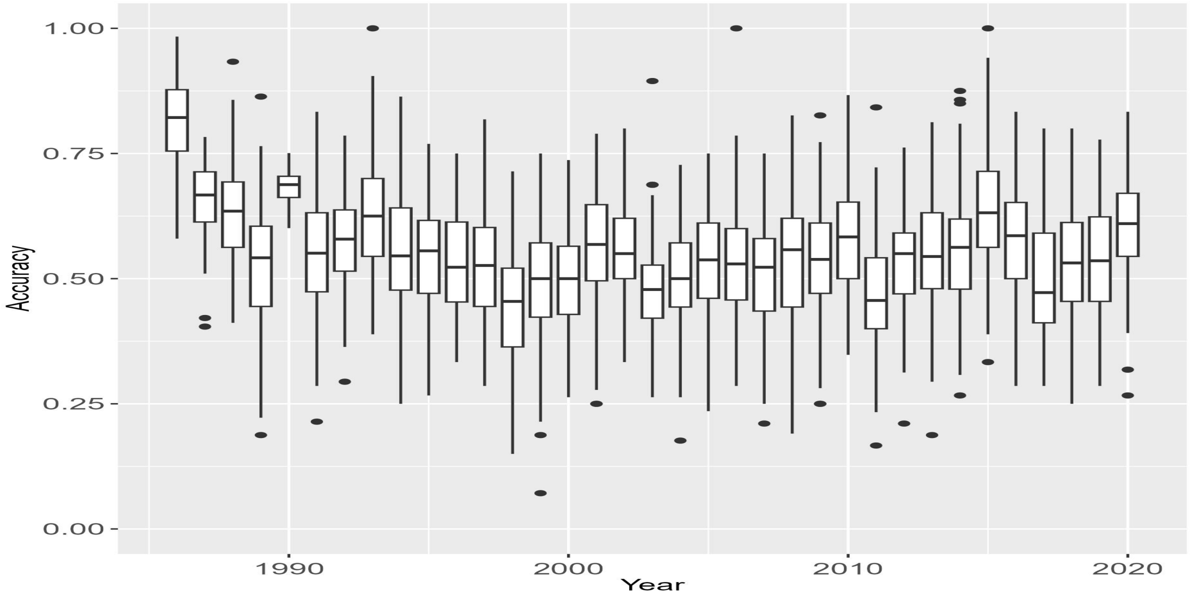

Figure 12. The boxplot illustrates the accuracy of 100 iterations of the random forest model for each year. The random forest model was applied to the communities detected by the InfoMap procedure on the network G_t^W. In each iteration, we sampled the training dataset and measured the accuracy on the test set.

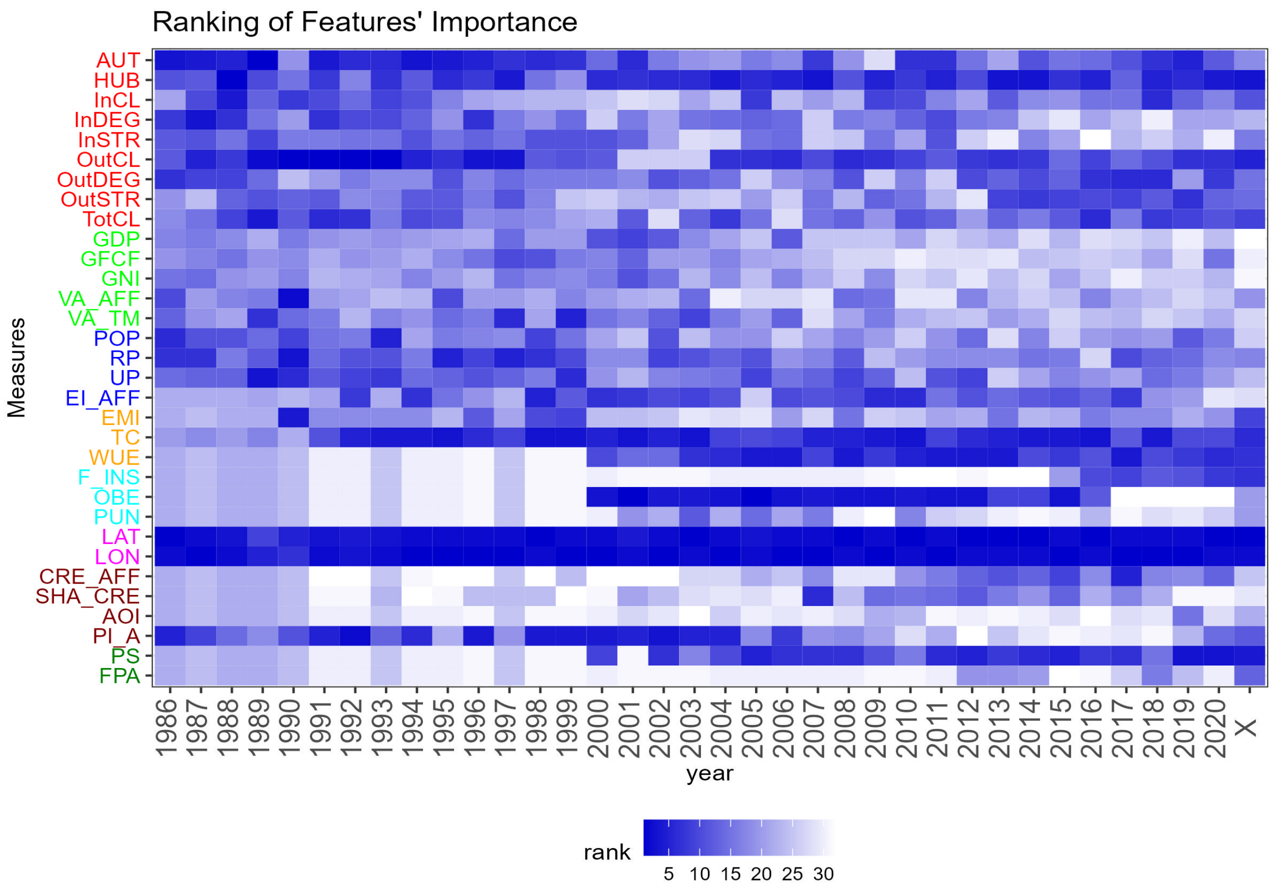

Figure 13. The ranking of features' importance over time is based on Mean Decrease Accuracy, using communities detected on the networks G_t^W. Darker blue indicates a higher rank. The y-axis labels of features have been grouped and coloured according to macro-categories. From top to bottom, the categories are: Topological Indicators, Macro Economic, Population and Employment, Climate and Sustainability, Food Security and Nutrition, Geographical, Investment and Production, and Others.

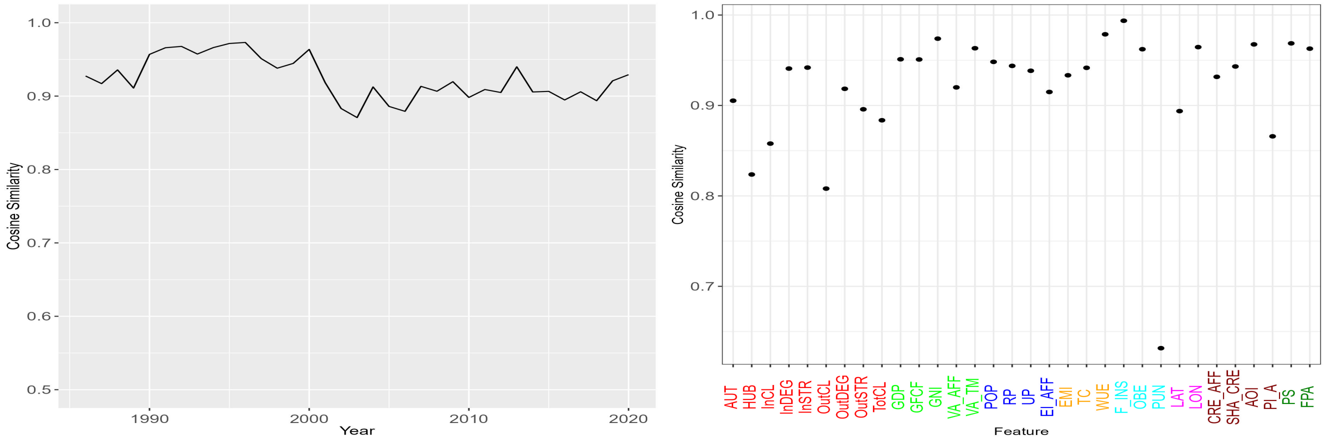

(a) For each year, we present the cosine similarity between the ranks of features based on networks where weights are represented by monetary values and quantities.

(b) For each feature, we show the cosine similarity between its ranks over time, based on networks where weights are represented by monetary values and quantities.

Figure 14. Cosine Similarity between ranks across years or features.

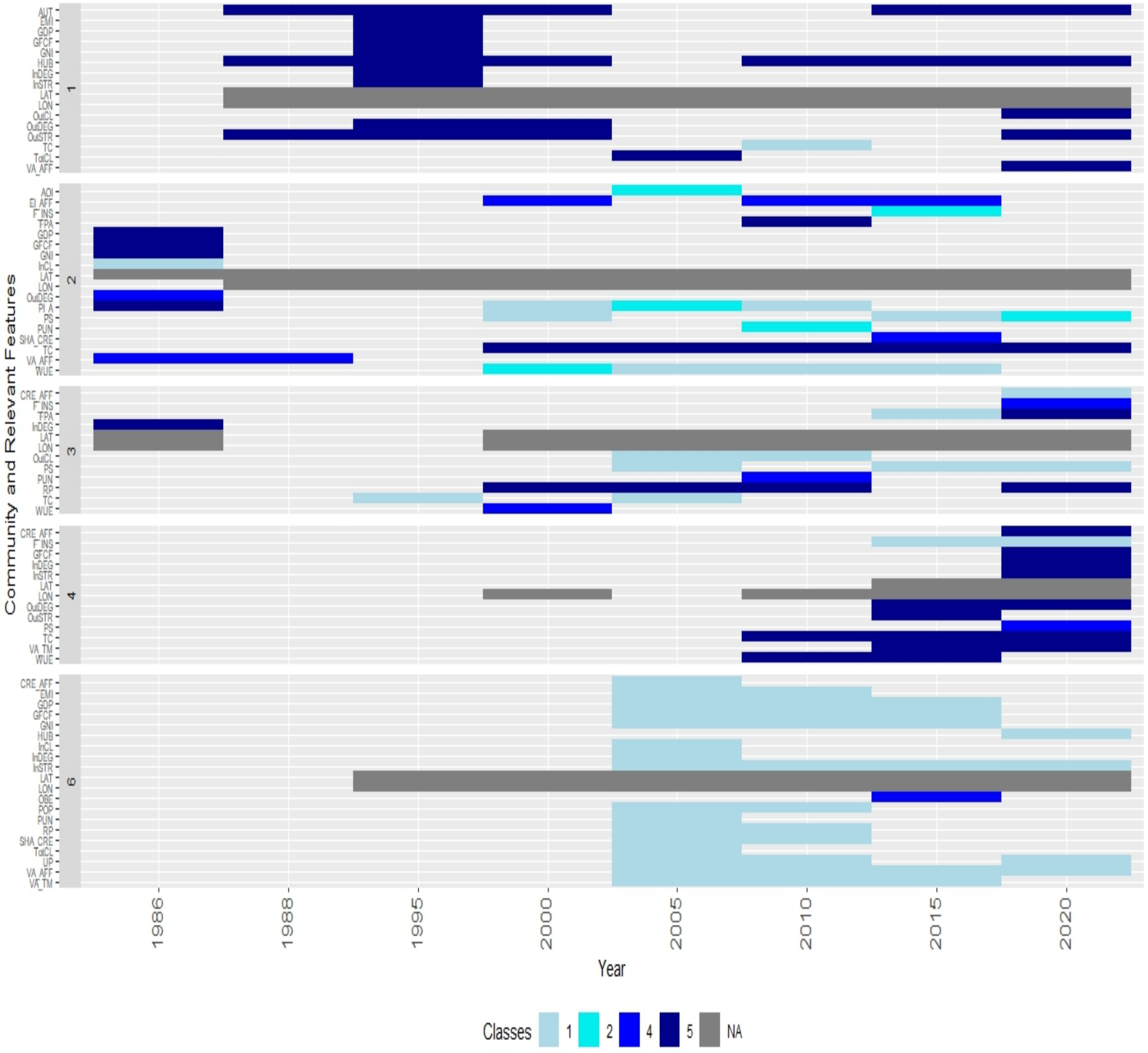

Figure 15. We present the features and their corresponding significant classes for the main communities based on networks G_t. The classes are labelled from 1 to 5 and defined as follows: $[0, q_{20\%}]$, $(q_{20\%} - q_{40\%}]$, $(q_{40\%} - q_{60\%}]$, $(q_{60\%} - q_{80\%}]$, $(q_{80\%} - q_{100\%}]$, where q_α represents the quantile of order α of the empirical values of the variable. For latitude and longitude, classes are not reported as they are not highly relevant in terms of interpretation. To ensure consistent comparison and maintain coherence with other figures, we used the following classification for this figure: Community 1 - includes North America, China, Japan, and Australia; Community 2 - includes Russia and neighbouring countries; Community 3 - includes Middle East; Community 4 - includes Europe; Community 6 - includes Central America and Venezuela.

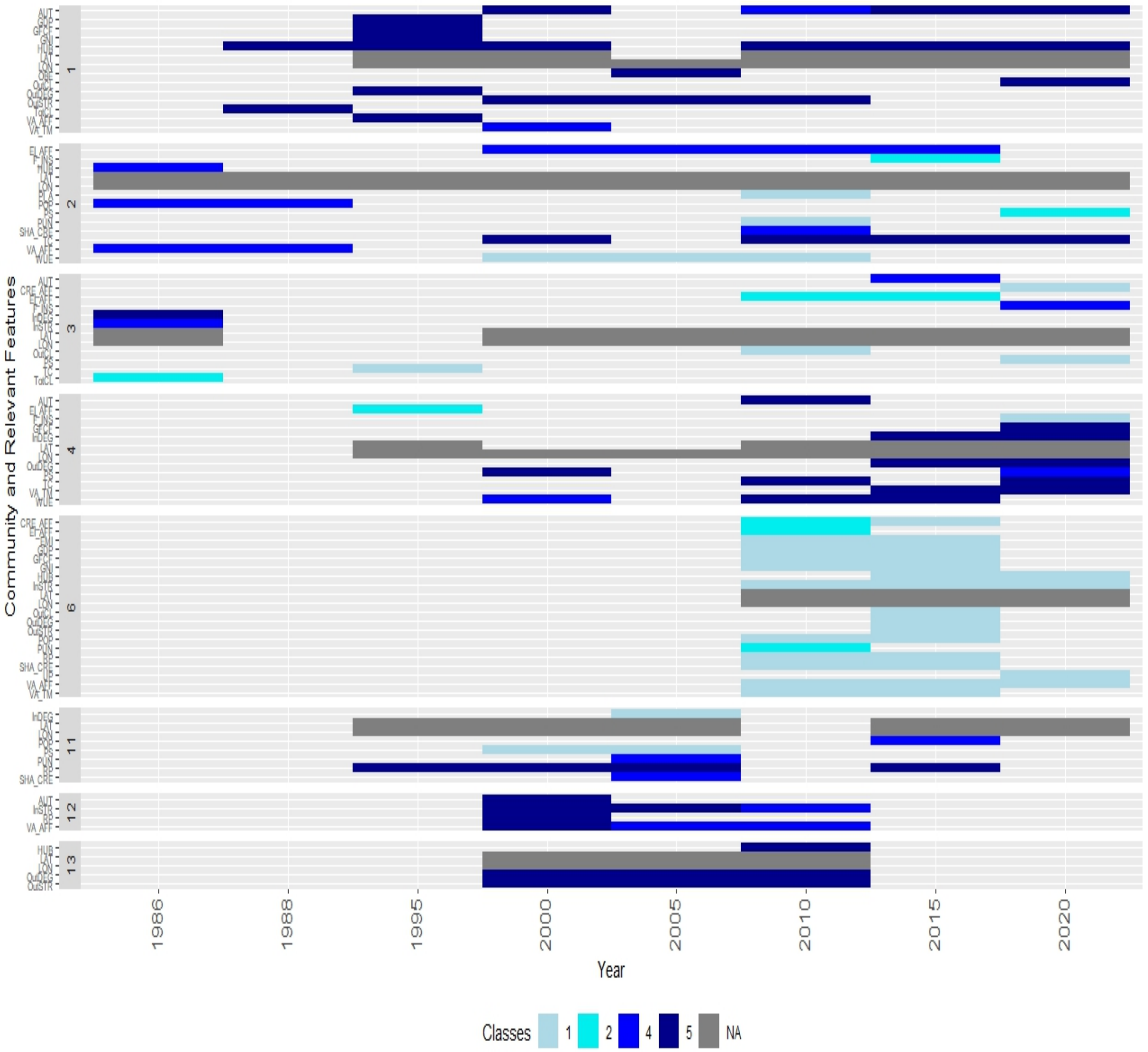

Figure 16. We present the features and their corresponding significant classes for the main communities based on networks $G^W t$. The classes are labelled from 1 to 5 and defined as follows: $[0, q_{20\%}]$, $(q_{20\%} - q_{40\%}]$, $(q_{40\%} - q_{60\%}]$, $(q_{60\%} - q_{80\%}]$, $(q_{80\%} - q_{100\%}]$, where q_α represents the quantile of order α of the empirical values of the variable. For latitude and longitude, classes are not reported as they are not highly relevant in terms of interpretation. Since the community varies over time, to ensure a consistent comparison and maintain coherence with other figures, we used the following classification in this figure: Community 1 - includes North America, China, Japan, and Australia, Community 2 - includes Russia and neighbouring countries, Community 3 - includes the Middle East, Community 4: includes Europe, Community 6 - includes Central America and Venezuela, Community 11 - includes India and Nepal for years (1995 to 2005, 2015, 2020) when they are not included in Community 3, Community 12 - includes China, Japan, and Australia for years (2000 to 2010) when they are not included in Community 1, Community 13 - includes Argentina and Brazil for years (2000 to 2010) when they are not included in the previous communities.

Communities	Macroarea/Countries	Relevant Features
1	North and South America, Australia<br>New Zealand, China, Japan, ASEAN countries	AUT(5), HUB(5), OutCL(5), OutSTR(5),<br>VA_AFF(5), LAT, LON
2	Russia, Turkiye, Ukraine, Central Asia, Caucasus	TC(5), LAT, LON, PS(2)
3	Middle East, India, Pakistan, Guinea, Gambia, Benin	RP(5), F_INS(4)<br>LAT, LON, CRE_AFF(1,2), PS(1), FPA(5)
4	Europe, Morocco, Algeria, Tunisia,<br>Guinea, Sierra Leone,<br>Islands (Polynesia, New Caledonia, Solomon)	InDEG(4,5), InSTR(5), OutDEG(4,5),<br>GFCF(5), VA_TM(5), TC(5), F_INS(1),<br>LAT, LON, CRE_AFF(5), PS(4)
5	Antigua and Barbuda, Mali, Mauritania, Senegal	LAT, LON
6	Central America, Venezuela	InSTR(1), HUB(1,2), VA_AFF(1)<br>UP(1), LAT, LON
7	South Africa and neighbouring countries	LAT, LON
8	Burundi, Democratic Republic of the Congo, Kenya, Malawi,<br>Rwanda, Uganda, United Republic of Tanzania, Zambia	LAT, LON
9	Fiji, Tuvalu	LAT, LON

Table 3. We report the composition of communities in 2020 for the network based on monetary value of trade, along with the significant variable for each community. The corresponding class of the variable is shown in brackets, labelled from 1 to 5 and defined as follows: $[0, q_{20\%}]$, $(q_{20\%} - q_{40\%}]$, $(q_{40\%} - q_{60\%}]$, $(q_{60\%} - q_{80\%}]$, $(q_{80\%} - q_{100\%}]$, where q_α represents the quantile of order α of the empirical values of the variable. Variables are coloured based on the macro-category, as in the previous plots.

Communities	Macroarea/Countries	Relevant Features
1	North and South America, Australia,<br>New Zealand, China, Japan, ASEAN countries	AUT(5), HUB(5), OutCL(5),<br>LAT, LON
2	Russia, Turkiye, Ukraine, Caucasus	TC(5), LAT, LON, PS(2)
3	Middle East	F_INS(4), LAT, LON<br>CRE_AFF(1,2), PS(1)
4	Europe, Morocco, Algeria, Tunisia, Sierra Leone<br>Islands (Polynesia, New Caledonia, Solomon)	InDEG(5), OutDEG(5), GFCF(5), VA_TM(5)<br>TC(5), F_INS(1), LAT, LON, PS(4)
5	Antigua and Barbuda, Cote d'Ivorie Ghana, Mali,<br>Mauritania, Niger, Nigeria, Senegal, Togo	LAT, LON
6	Central America, Venezuela	InSTR(1), HUB(1,2), VA_AFF(1)<br>UP(1), LAT, LON
7	South Africa and neighbouring countries	LAT, LON
8	Burundi, Democratic Republic of the Congo, Kenya,<br>Rwanda, Uganda, United Republic of Tanzania	LAT, LON
9	Fiji, Tuvalu, Kiribati	LAT, LON
10	Central Asia, Pakistan	LAT, LON
11	Benin, Gambia, Guinea, India, Nepal	LAT, LON
12	Madagascar	

Table 4. We report the composition of communities in 2020 for the network based on volume of trade in tonnes, along with the significant variable for each community. The corresponding class of the variable is shown in brackets, labelled from 1 to 5 and defined as follows: $[0, q_{20\%}]$, $(q_{20\%} - q_{40\%}]$, $(q_{40\%} - q_{60\%}]$, $(q_{60\%} - q_{80\%}]$, $(q_{80\%} - q_{100\%}]$ where q_α represents the quantile of order α of the empirical values of the variable. Variables are coloured based on the macro-category, as in the previous plots.

References

1. R Core Team. *R: A Language and Environment for Statistical Computing*. R Foundation for Statistical Computing, Vienna, Austria (2021).
2. Newman, M. E. J. The structure and function of complex networks. *SIAM Rev.* **6**, 28384, DOI: 10.1038/srep28384 (2003).
3. Fagiolo, G. Clustering in complex directed networks. *Phys. Rev. E* **76** (2007).
4. Clemente, G. P. & Grassi, R. Directed clustering in weighted networks: A new perspective. *Chaos, Solitons & Fractals* **107**, 26–38 (2018).
5. Kleinberg, J. M. Authoritative sources in a hyperlinked environment. *J. ACM (JACM)* **46**, 604–632 (1999).
6. FAO. FAOSTAT, FAO Statistical Database. Tech. Rep., Food and Agriculture Organization of the United Nations (2023).
